# No effects without causes: the Iron Dysregulation and Dormant Microbes hypothesis for chronic, inflammatory diseases

**DOI:** 10.1111/brv.12407

**Published:** 2018-03-25

**Authors:** Douglas B. Kell, Etheresia Pretorius

**Affiliations:** ^1^ School of Chemistry The University of Manchester, 131 Princess Street Manchester Lancs M1 7DN U.K.; ^2^ The Manchester Institute of Biotechnology The University of Manchester, 131 Princess Street Manchester Lancs M1 7DN U.K.; ^3^ Department of Physiological Sciences Stellenbosch University, Stellenbosch Private Bag X1 Matieland 7602 South Africa

**Keywords:** amyloid, inflammation, iron dysregulation, blood clotting, LPS, amplification

## Abstract

Since the successful conquest of many acute, communicable (infectious) diseases through the use of vaccines and antibiotics, the currently most prevalent diseases are chronic and progressive in nature, and are all accompanied by inflammation. These diseases include neurodegenerative (e.g. Alzheimer's, Parkinson's), vascular (e.g. atherosclerosis, pre‐eclampsia, type 2 diabetes) and autoimmune (e.g. rheumatoid arthritis and multiple sclerosis) diseases that may appear to have little in common. In fact they all share significant features, in particular chronic inflammation and its attendant inflammatory cytokines. Such effects do not happen without underlying and initially ‘external’ causes, and it is of interest to seek these causes. Taking a systems approach, we argue that these causes include (i) stress‐induced iron dysregulation, and (ii) its ability to awaken dormant, non‐replicating microbes with which the host has become infected. Other external causes may be dietary. Such microbes are capable of shedding small, but functionally significant amounts of highly inflammagenic molecules such as lipopolysaccharide and lipoteichoic acid. Sequelae include significant coagulopathies, not least the recently discovered amyloidogenic clotting of blood, leading to cell death and the release of further inflammagens. The extensive evidence discussed here implies, as was found with ulcers, that almost all chronic, infectious diseases do in fact harbour a microbial component. What differs is simply the microbes and the anatomical location from and at which they exert damage. This analysis offers novel avenues for diagnosis and treatment.

‘*The great enemy of truth is very often not the lie – deliberate, contrived and dishonest – but the myth – persistent, persuasive and unrealistic. Too often we hold fast to the clichés of our forebears. We subject all facts to a prefabricated set of interpretations. We enjoy the comfort of opinion without the discomfort of thought*’. John F. Kennedy, Commencement Address, Yale University, June 11 1962

‘*These germs ‐ these bacilli ‐ are transparent bodies. Like glass. Like water. To make them visible you must stain them. Well, my dear Paddy, do what you will, some of them won't stain; they won't take cochineal, they won't take any methylene blue, they won't take gentian violet, they won't take any colouring matter. Consequently, though we know as scientific men that they exist, we cannot see them*’. Sir Ralph Bloomfield‐Bonington. *The Doctor's Dilemma*. George Bernard Shaw, 1906.

## I. INTRODUCTION

A very large number of chronic, degenerative diseases are accompanied by inflammation. Many of these diseases are extremely common in the modern ‘developed’ world, and include vascular (e.g. atherosclerosis, type 2 diabetes, metabolic syndrome, pre‐eclampsia, stroke), autoimmune [e.g. rheumatoid arthritis (RA), multiple sclerosis], and neurodegenerative (e.g. Alzheimer's, Parkinson's, Amyotrophic lateral sclerosis) diseases. On the face of it these diseases are quite different from each other, but in fact they share a great many hallmarks [and often comorbidities (see e.g. Agustí & Faner, 2012; Altamura & Muckenthaler, 2009; Figueira *et al.,* 2016; Lago *et al.,* 2011; Nanhoe‐Mahabier *et al.,* 2009; Pretorius, Mbotwe & Kell, 2017b; Shen *et al*., 2016)]. As well as inflammation, these hallmarks include increased levels of inflammatory cytokines (almost a definition of inflammation), dysregulation in iron metabolism [especially the appearance of abnormal levels of ferritin in the serum (Kell & Pretorius, 2014)], and a variety of coagulopathies and haematological pathologies (abnormalities in the blood system, including its clotting properties). Many of these diseases also share other properties such as an increase in ‘insoluble’ forms of normally soluble proteins and of microparticulate material. Although they are progressive diseases, their progress is far from uniform, and they are often accompanied by fluctuating changes in physiological states (such as ‘flares’ in rheumatoid arthritis).

However, these ‘hallmarks’ are effectively physiological biomarkers; they are responses to one or more initial external stimuli, and they can and do serve as mediators for (later) manifestations of overt disease. Since effects do not happen without causes, however, the question then arises as to the identity of these external stimuli. In some cases (especially atherosclerosis and metabolic syndrome) there is evidence for a significant dietary component. However, based on a now considerable and wide‐ranging literature, we here bring together evidence that: (*i*) the main external stimuli are microorganisms; (*ii*) in contrast to what happens in conventional infectious diseases they do not proliferate unchecked, but commonly enter dormant states that make them invisible to classical microbiology; and (*iii*) they can be reactivated from these dormant states by the presence of ‘free’ iron (a necessary nutrient that in unliganded form is normally at low levels in the host). This reactivation releases highly potent inflammagens such as lipopolysaccharide (LPS) from Gram‐negative organisms and lipoteichoic acid (LTA) from Gram‐positives. Various sequelae, including coagulopathies, amyloid formation and cell death follow from this, and thus we argue that this general explanation – that we refer to here as the Iron Dysegulation and Dormant Microbes (IDDM) hypothesis–underpins a host of these chronic, inflammatory diseases.

**Figure 1:**
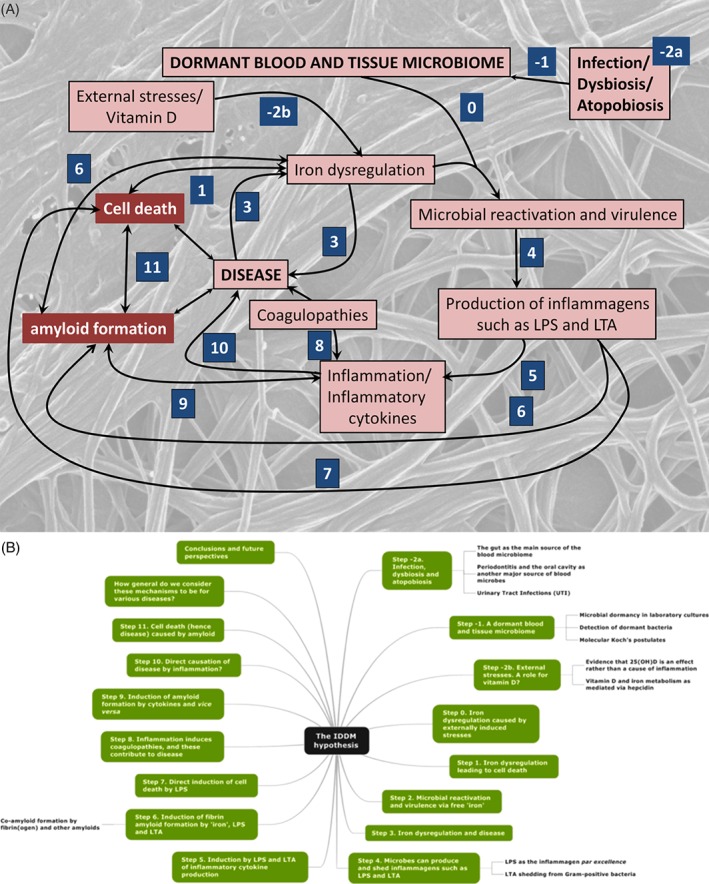
Overview of the processes involved in the Iron Dysregulation and Dormant Microbes (IDDM) hypothesis of chronic inflammatory diseases. (A) The numbered steps, starting with steps –2a and –2b, that are discussed sequentially in this review. (B) A ‘mind map’ (Buzan, 2002) of this review. LPS, lipopolysaccharide; LTA, lipoteichoic acid; 25(OH)D_3_, 25‐hydroxy‐D_3_ (vitamin D).

As discussed previously (Kell, 2006; Kell & Knowles, 2006), a typical systems biology strategy (Alon, 2006; Klipp *et al*., 2005; Palsson, 2006) consists of several phases. The first is qualitative, in which we identify the main players and the main interactions among them. This is the ‘curly arrow’ version that sets out the system of interest in the form of a ‘graph’ containing nodes (players) and edges (their interactions). The nodes can be high level, e.g. processes, or lower level (e.g. individual enzymes in a network). Later steps may seek to become quantitative in the sense that we provide equations for the interactions and then seek to parameterise them (Maldonado *et al*., 2017). At present, we are still at the very first step or highest level, i.e. providing only the ‘curly arrow’ diagram. We are not yet even in a position to follow good practice (Le Novère *et al*., 2009) by discriminating the types of interaction by changing the graphical notation. Fig. 1 sets out the main steps involved, and summarises this review in the form of a ‘mind map’. Note, however, that while for convenience we have separated the various steps, some are contemporaneous, and a variety of other interactions and feedbacks are omitted for clarity of presentation. The main focus of this review is the evidence for each of the steps outlined in Fig. 1A.

## II. STATE –2A: INFECTION, DYSBIOSIS AND ATOPOBIOSIS

While microbiomes such as the skin microbiome (Dréno *et al*., 2016; Dybboe *et al*., 2017; Edmonds‐Wilson *et al*., 2015; Fitz‐Gibbon *et al*., 2013; Kong *et al*., 2017; Kong *et al*., 2012; Oh *et al*., 2016, 2013; SanMiguel & Grice, 2015; van Rensburg *et al*., 2015) and the gut microbiome (see Section II.1) are well known, many other sites that are widely considered sterile are in fact full of microbes (Bullman, Meyerson & Kostic, 2017; Ding & Schloss, 2014; Foster *et al*., 2017; Garn *et al*., 2016; The Human Microbiome Project Consortium, 2012; Lloyd & Marsland, 2017; Lluch *et al*., 2015). As well as blood, which we also discuss in detail herein, these include the respiratory system (e.g. Bassis *et al*., 2015; Budden *et al*., 2017; Dickson *et al*., 2017, 2016, *b*; Dickson & Huffnagle, 2015; Huffnagle, Dickson & Lukacs, 2017; O'Dwyer, Dickson & Moore, 2016; Samuelson, Welsh & Shellito, 2015; Vientós‐Plotts *et al*., 2017, *b*), neck tissue (Wang *et al*., 2017), breast tissue (Wang *et al*., 2017), and both seminal fluid (Craig *et al*., 2015; Hou *et al*., 2013; Javurek *et al*., 2016; Kenny & Kell, 2018; C.M. Liu *et al*., 2014; Mändar *et al*., 2015; Weng *et al*., 2014) and the placenta (Aagaard *et al*., 2014; Amarasekara *et al*., 2015; Antony *et al*., 2015; Collado *et al*., 2016; Pelzer *et al*., 2016; Prince *et al*., 2016; Tarazi, Agostoni & Kim, 2014; Zheng *et al*., 2015) (*cf*. Lauder *et al*., 2016). Indeed, probably all tissues harbour fairly considerable numbers of non‐growing microbes even under normal conditions (Bullman *et al*., 2017; Domingue, Turner & Schlegel, 1974; Domingue, 2010; Domingue & Woody, 1997; Gargano & Hughes, 2014; Mattman, 2001; Proal, Albert & Marshall, 2013, 2014; Proal, Lindseth & Marshall, 2017).

### (1) The gut as the main source of the blood microbiome

We are surrounded by microbes, and are exposed to them constantly. In particular, the gut microbiome has attracted considerable attention, as the number of microbial cells it harbours is similar to or even greater than those in the human body – some 10^13^ to 10^14^ (Chu & Aagaard, 2016; Charbonneau *et al*., 2016; Foster *et al*., 2017; Guinane & Cotter, 2013; Mondot *et al*., 2013; Noecker *et al*., 2017; Turnbaugh *et al*., 2007; Walter & Ley, 2011). Recent developments include the recognition that many of the soluble metabolic products of the gut microbiome can enter the bloodstream, and hence circulate throughout the body (Dodd *et al*., 2017; Schroeder & Bäckhed, 2016), including to the central nervous system (CNS) where they can have profound neurological effects. This is known as the ‘gut–brain axis’ (e.g. Alonso *et al*., 2014; Houser & Tansey, 2017; Montiel‐Castro *et al*., 2013; Sandhu *et al*., 2017; Schroeder & Bäckhed, 2016; Sherwin, Dinan & Cryan, 2017). Large amounts of insoluble LPS are also present in the gut (∼1 g; Zaman & Zaman, 2015), and these too can pass into the bloodstream (de Punder & Pruimboom, 2015; Kell & Pretorius, 2015a; Maes, Coucke & Leunis, 2007).

Almost everything dietary, including medicines (Le Bastard *et al*., 2017), can affect the gut microbiome [and *vice versa* (Gillis *et al*., 2018; Koppel, Maini Rekdal & Balskus, 2017; Wilson & Nicholson, 2017)], and there is a large literature, that we do not seek to summarise (Subramanian *et al*., 2015), on the use of prebiotics and probiotics that are intended to modify it. There is consequently no such thing as a or the ‘normal’ gut microbiome, although certain patterns or frequencies of microbial types are seen as representing some kind of commonality (Lloyd‐Price *et al*., 2017), at least to the ethnic group under study. For our purposes, the main significance is that the gut microbiome is large and that it exists. ‘Dysbiosis’ is a term usually used to mean a change in the gut microbiome such that its composition differs significantly from those of the ‘normal’ (commonest) populations of interest (Olesen & Alm, 2016) and we adopt this usage herein. Unfortunately, ‘dysbiosis’ is also used, misleadingly, to refer to the appearance of gut microbes in other places; we have therefore suggested the use of the word ‘atopobiosis’ for this latter meaning [microbes in the ‘wrong’ place (Potgieter *et al*., 2015)].

Inevitably, some of these microbes can display atopobiosis, and enter the bloodstream from the gut (de Punder & Pruimboom, 2015; van der Meulen *et al*., 2016). When this influx is particularly great, it is sometimes referred to as a ‘leaky gut’ (e.g. Fasano, 2012; Kato *et al*., 2017; Li & Atkinson, 2015; Luettig *et al*., 2015; Maes, 2009; Maes *et al*., 2007; Mu *et al*., 2017; Quigley, 2016; Shukla *et al*., 2015; Thevaranjan *et al*., 2017; Wallace *et al*., 2014). The result of this, and of the two other main sources that we cover in Sections II.2 and III.3, is the existence of a standing crop of microbes that have entered the bloodstream (Kell, Potgieter & Pretorius, 2015; Potgieter *et al*., 2015). Fortunately, they do not normally lead to bacteraemia in the form of readily culturable, replicating microbes, as this could be extremely serious (Havey, Fowler & Daneman, 2011; Holland, Arnold & Fowler Jr, 2014; Versalovic *et al*., 2011; Wester *et al*., 2014).

### (2) Periodontitis and the oral cavity as another major source of blood microbes

A second common origin for blood microbes is the non‐sterile oral cavity (Gargano & Hughes, 2014), whence they can enter through abrasive toothbrushing (Bhanji *et al*., 2002; Tomás *et al*., 2012) or periodontal disease. Since blood can appear in the oral cavity, there is nothing to stop the reverse process of microbial infection of the blood (Dhotre, Davane & Nagoba, 2017; Kilian *et al*., 2016; Koren *et al*., 2011) and periodontal origins represent another source of potential microbial translocation (Moon & Lee, 2016). There is considerable evidence for a significant association between periodontitis and RA (Bingham III & Moni, 2013; Cheng *et al*., 2018; de Smit *et al*., 2012; Detert *et al*., 2010; Konig *et al*., 2016; Koziel, Mydel & Potempa, 2014; Lee *et al*., 2015; Martinez‐Martinez *et al*., 2009; Mikuls *et al*., 2009; Monsarrat *et al*., 2013; Ogrendik, 2013; Potempa, Mydel & Koziel, 2017). Atherosclerosis provides another example (Chukkapalli *et al*., 2015; Gibson III & Genco, 2007; Kebschull, Demmer & Papapanou, 2010; Łysek *et al*., 2017; Mahalakshmi *et al*., 2017; Rangé *et al*., 2014; Reyes *et al*., 2013; Rivera *et al*., 2013; Teeuw *et al*., 2014; Toyofuku *et al*., 2011; Velsko *et al*., 2014).

### (2) Urinary tract infections (UTIs)

While any location of an infection, e.g. the chest, is a potential source of microbes that could enter the bloodstream, the other main source of microbial infections of present interest is probably the urinary tract (Flores‐Mireles *et al*., 2015). For anatomical reasons, women are some 3.5 times more likely to suffer UTIs than are men, an infection that returns frequently because it is not completely eradicated (Blango & Mulvey, 2010; Blango *et al*., 2014; Ejrnæs, 2011; Hannan *et al*., 2012; Mysorekar & Hultgren, 2006; Pretorius *et al*., 2017a; Rosen *et al*., 2007; Schwartz *et al*., 2011); this brings us to the physiological state of the bacteria involved. While most would agree with the idea that certain clades of bacteria regularly enter dormant or latent states, not least *Mycobacterium tuberculosis* (Alnimr, 2015; Barry III *et al*., 2009; Chao & Rubin, 2010; Gengenbacher & Kaufmann, 2012), which can remain inactive in the lungs for decades, the idea that this may actually be the norm has not yet taken hold.

## III. STEP –1: A DORMANT BLOOD AND TISSUE MICROBIOME

The chief method of classical microbiology involves plating a suitably diluted subsample from the sample of interest onto a ‘solid’ (usually agar) medium considered likely to allow their proliferation, and waiting until visible colonies are formed, the number of ‘colony‐forming units’ (CFUs) being equal to the number of ‘viable’ bacteria in the subsample. There are numerous growth media [the classic listing (Zimbro *et al*., 2009) runs to 700 pages], and typically rather ‘rich’ media are used. One such medium, known euphemistically as ‘chocolate’ agar, is based on blood that has been heated to 80°C to lyse erythrocytes. The concept that ‘viability’ = culturability, or the ability to replicate, is thus a cornerstone of microbiology (Postgate, 1967, 1969, 1976).

The problem with this general strategy is that not only are individual media not suitable for all organisms, but that most organisms (especially when starved) can enter physiological states in which rich media either do not support their growth or may actually kill them (and clearly it is hard to discriminate between these possibilities). However, the organisms may not be ‘dead’, as other treatments can restore them to a physiological state in which they do produce colonies on the same media. Under these circumstances we should refer to them as ‘dormant’ (Kaprelyants, Gottschal & Kell, 1993) since clearly they are not ‘dead’ – a state we take on classical semantic grounds to be irreversible. Dormancy, and any other physiological state, is then to be seen not as a property of the organism alone, but of the organism plus the test used to assess it, and thus these definitions are operational definitions (Kell *et al*., 1998), reflecting the ‘Schrödinger's cat' problem of quantum mechanics (Primas, 1981).

Indeed, in nature, dormancy is in fact the norm (e.g. Buerger *et al*., 2012; Dworkin & Shah, 2010; Jones & Lennon, 2010; Kell *et al*., 2015; Kell & Pretorius, 2015a; Lennon & Jones, 2011; Lewis, 2007; Potgieter *et al*., 2015; Rittershaus, Baek & Sassetti, 2013; Sachidanandham & Yew‐Hoong Gin, 2009; Sturm & Dworkin, 2015; G.S. Wang *et al*., 2015a, 2014; Wood, Knabel & Kwan, 2013). This should be seen as rather unsurprising, in that it is reasonable that organisms evolved (or were selected) such that when they ran out of essential nutrients or necessary signalling molecules, and could not replicate, they did not simply die but entered some kind of dormant state from which they might be resuscitated in better times (Mukamolova *et al*., 2003). In clinical microbiology, the term ‘persistence’ (Balaban *et al*., 2013; Cohen, Lobritz & Collins, 2013; Dehio, Berry & Bartenschlager, 2012; Fauvart, De Groote & Michiels, 2011; Gerdes & Maisonneuve, 2012; Harms, Maisonneuve & Gerdes, 2016; Holden, 2015; Kester & Fortune, 2014; Krebs, Bartel & Pannek, 2014; Lewis, 2007, 2010; Orman & Brynildsen, 2013; Shah *et al*., 2006; Wood *et al*., 2013; Y. Zhang, Yew & Barer, 2012) has come to mean operationally the same thing, i.e. a phenotypic (non‐genotypic) reversible change to an apparently non‐culturable state. In clinical settings, this is often in the presence of otherwise toxic concentrations of antibiotics, where the adoption of a dormant or ‘persistent’ state permits survival.

We note that the term ‘viable‐but‐not‐culturable’ has been used occasionally, despite the fact that this is an oxymoron if one accepts that viability = culturability. Although it is starting to be recognised that microbes said to be adopting this state may in fact be dormant (Oliver, 2010), we suggest that the term ‘viable‐but‐not‐culturable’ is avoided altogether (Kell *et al*., 1998).

### (1) Microbial dormancy in laboratory cultures

A clear‐cut demonstration of dormancy under controlled, laboratory conditions, came from studies of *Micrococcus luteus* performed in the 1990s. Briefly, starvation after batch culture led to a loss of culturability to approximately 10^−3^ to 10^−5^ of the total cell count (Kaprelyants & Kell, 1992, 1993), accompanied by anticipated morphological and biochemical changes, including the conversion of most lipids to cardiolipin (Mukamolova *et al*., 1995). However, the cells could be resuscitated in the presence of spent culture supernatant under conditions of dilution to extinction (Kaprelyants, Mukamolova & Kell, 1994; Votyakova, Kaprelyants & Kell, 1994). The active constituent in this supernatant was a protein (Mukamolova *et al*., 1998) with a specific resuscitation promotion factor (Rpf) motif that is present in a wide range of actinobacteria (Mukamolova *et al*., 1999, 2006, 2002, *b*). These features were recognised (Mukamolova *et al*., 2003) as an important survival strategy. The importance of the ‘dilution to extinction’ experiments was that they avoided any confounding effect of small numbers of ‘actually viable’ cells that could simply regrow and/or resuscitate others. Specifically, resuscitation of the dormant cells was enhanced considerably by an initial period of incubation in weak nutrient broth.

### (2) Detection of dormant bacteria

Were the microbes that enter the blood to be capable of replicating in a medium that – like ‘chocolate’ agar – is actually quite rich in organic molecules, we would be discussing conventional, infectious diseases and bacteraemia as commonly understood, but we are not. Under normal conditions, however, either because of the innate immune system or the physiological state of the microbes, or both, normal (non‐bacteraemic) blood – as judged by classical microbiological criteria – is indeed sterile, i.e. it is not possible to detect the presence of viable bacteria in this way. To investigate whether dormant bacteria are present, we thus need culture‐independent methods, of which ultramicroscopic (e.g. Domingue *et al*., 1974; Domingue, 1995, 2010; Domingue & Woody, 1997; Ewald, 2002; Green, Heidger Jr & Domingue, 1974a,*b*; Mattman, 2001; Potgieter *et al*., 2015) and molecular sequence‐based methods (Amar *et al*., 2011; Cherkaoui *et al*., 2009; Fernández‐Cruz *et al*., 2013; Gaibani *et al*., 2013; Grif *et al*., 2012,*b*; C.L. Liu *et al*., 2014; Moriyama *et al*., 2008; NIH HMP Working Group *et al*., 2009; Nikkari *et al*., 2001; Sakka *et al*., 2009; Sato *et al*., 2014; Valencia‐Shelton & Loeffelholz, 2014; Woyke, Doud & Schulz, 2017) are by far the most common.

We also recognise that dormant bacteria can survive in white blood cells (Liehl, Zuzarte‐Luis & Mota, 2015; Miskinyte & Gordo, 2013; Miskinyte *et al*., 2013; Ribet & Cossart, 2015; Thwaites & Gant, 2011), and probably also in the much more prevalent red blood cells (Potgieter *et al*., 2015), just as can classically infectious organisms such as *Bartonella* spp. (Ben‐Tekaya, Gorvel & Dehio, 2013; Dehio, 2001; Eicher & Dehio, 2012; Pitassi *et al*., 2007; Seubert, Schulein & Dehio, 2002), *Francisella tularensis* (Conlan, 2011; Horzempa *et al*., 2011), various mycoplasmas (e.g. Groebel *et al*., 2009), and *Streptococcus pneumoniae* (Yamaguchi *et al*., 2013).

A large number of studies (e.g. Domingue *et al*., 1974; Domingue *et al*., 1995; Domingue & Schlegel, 1977a,*b*; Domingue & Woody, 1997; Goubran *et al*., 2017; Mattman, 2001; Nikkari *et al*., 2001), reviewed previously by Amar *et al*. (2011), Kell & Kenny (2016), Kell *et al*. (2015); Kell & Pretorius (2015a) and Potgieter *et al*. (2015), suggests that there is indeed an authentic but dormant blood microbiome. A particularly good example comes from Damgaard *et al*. (2015) who reasoned that plating samples from blood bags straight onto chocolate agar exposed them to atmospheric oxygen, and that this might produce reactive oxygen species that could kill any organisms present. When instead they plated them anaerobically, the supposedly sterile blood revealed a large resident microbiome that could be cultured (and indeed sequenced). Many microbes resident in humans are as yet uncharacterised (Kowarsky *et al*., 2017), and evolutionary arguments support the idea that it is often better to tolerate than to fight against invading organisms (Ayres, 2016; Ayres & Schneider, 2012; Schneider & Ayres, 2008).

In particular, those recognising relationships between overt chronic, inflammatory disease and the presence of detectable microbes, can highlight that the blood and tissue microbiome is greatly enhanced in these diseases (Alonso *et al*., 2017; Arleevskaya *et al*., 2016; Berstad & Berstad, 2017; Broxmeyer, 2017a,*b*; Ebringer, 2012; Ebringer & Rashid, 2009; Ebringer, Rashid & Wilson, 2010; Emery *et al*., 2017; Itzhaki *et al*., 2016; Kell & Kenny, 2016; Maheshwari & Eslick, 2015; Miklossy, 2011; Miklossy & McGeer, 2016; Pisa *et al*., 2017; Pretorius *et al*., 2017a; Pretorius, Bester & Kell, 2016a; Proal *et al*., 2013, 2014, 2017). We note too that while it is all too easy to dismiss such findings as ‘contaminants’, those doing so must also explain why the microbes appear at much higher levels only in the ‘disease’ samples.

### (3) Molecular Koch's postulates

The Henle–Koch postulates (that microbe X causes disease Y) represent another cornerstone of infection microbiology (Autenrieth, 2016; Evans, 1976; Gradmann, 2014; Segre, 2013); they require association of the proposed pathogen with the disease and non‐association in its absence, as well as reinfection leading to renewed disease. Specifically, (*i*) the microorganism must be found in diseased but not in healthy individuals; (*ii*) the microorganism must be cultured from the diseased individual; (*iii*) inoculation of a healthy individual with the cultured microorganism must recapitulate the disease; and finally (*iv*) the microorganism must be reisolated from the inoculated, diseased individual and must match the original microorganism. Unfortunately these original concepts simply do not work in the case of dormant microbes (Antonelli & Cutler, 2016; Autenrieth, 2016; Byrd & Segre, 2016; Falkow, 1988, 2004; Fredricks & Relman, 1996; Seal *et al*., 2010), because it is not always possible to isolate culturable organisms from patients with the disease. In the case of Whipple's disease and the causative organism *Tropheryma whipplei*, a clear link between the disease and ultramicroscopically observable microbes was established (Maiwald & Relman, 2001; Relman *et al*., 1992) long before sequencing methods (Bentley *et al*., 2003) allowed the design of a medium in which the organism could be persuaded to replicate (Renesto *et al*., 2003). Thus, while the ideal would be the fulfilment of the original Koch's postulates, the association of specific DNA with the disease should nowadays be sufficient for the tentative identification of a causative organism even, as in the case of *H. pylori* and gastric ulcers (Marshall, 2001; Marshall *et al*., 1985, 1988), when an infectious agent was not previously suspected.

## IV. STEP –2B EXTERNAL STRESSES, AND A POSSIBLE ROLE FOR VITAMIN D

By our definition, causality demands an external stimulus. External stresses can be mechanical (e.g. trauma), oxidative, pharmacological or dietary [including poisoning (Kell, 2010)] among others. We here use an example of a dietary stimulus (vitamin D_3_) as an illustration of the complexity of the systems under discussion.

It has been pointed out previously (e.g. Mangin, Sinha & Fincher, 2014; Proal, Albert & Marshall, 2015) that vitamin D dysregulation is a common accompaniment to chronic infection with (normally) dormant microbes. Vitamin D dysregulation typically manifests as a low serum level of calcidiol [25‐hydroxy‐D_3_; 25(OH)D_3_] and is indeed widely observed in inflammation (Table 1), although whether it is a cause or a consequence cannot of course be determined from simple co‐occurrences. The studies listed in Table 1 show associations, but not (Beveridge & Witham, 2013; Cannell, Grant & Holick, 2014; Kienreich *et al*., 2013) whether low vitamin D levels are a cause or an effect of inflammation (or both, under different conditions; Cannell *et al*., 2014), how this relates to the disease, and whether improving some aspect of vitamin D status would be a treatment option.

**Table 1:** Chronic, inflammatory diseases in which low vitamin D levels have been recorded

Disease	Subtype	Comments	Reference
**‘Autoimmune’**		Review: strong inverse relationships between [25(OH)D_3_] and incidence of several automimmune diseases	Skaaby *et al*. (2015)
Chronic obstructive pulmonary disease (COPD)		Clear inverse relationship between COPD and vitamin D status	Skaaby *et al*. (2014)
Rheumatoid arthritis (RA)		Meta‐analysis of a large literature; mean [25(OH)D_3_] 16.5 nM lower in RA patients	Arnson *et al*. (2007); Lin *et al*., 2016)
**Cancer**	Multiple, especially skin	Acts with vitamin D receptor (VDR) *via* hedgehog and ß‐catenin	Bikle (2011)
	Skin	Role of ß‐catenin	Jiang *et al*. (2013)
		Meta‐analysis: little effect on incidence but significant effect on mortality	Keum & Giovannucci (2014)
	Multiple	Epidemiological	Afzal *et al*. (2014b)
**Cardiovascular**			
Atherosclerosis		Detailed reviews and meta‐analyses	Kassi *et al*. (2013); Menezes *et al*. (2014)
		Meta‐analysis	Carvalho & Sposito (2015)
Heart failure			de Temiño *et al*. (2011)
Hypertension			
		Odds ratio (OR) = 6.13 for incident hypertension in males if [25(OH)D_3_] <15 ng ml^−1^ *versus* ≥ 30 ng ml^−1^	Forman *et al*. (2007)
		OR = 1.66 for incident hypertension in lowest *versus* highest [25(OH)D_3_] quartile	Forman *et al*. (2008)
		Large meta‐analysis: 10% increase in [25(OH)D_3_] reduces hypertension risk by 8%; OR = 0.92	Vimaleswaran *et al*. (2014)
		Large meta‐analysis; risk ratio (RR) = 0.68 for highest *versus* lowest [25(OH)D_3_] category	Ke *et al*. (2015)
		Significantly lower, including in subsequent organ damage	Pludowski *et al*. (2014)
		OR = 13.54 for low [25(OH)D_3_] and risk of ischaemic stroke in hypertensives	Majumdar *et al*. (2015)
Myocardial infarction (MI) and cardiovascular disease		Epidemiological study; RR > 2 if [25(OH)D_3_] < 15 ng ml^−1^ (37 nM)	Giovannucci *et al*. (2008)
		Very large effects of low [25(OH)D_3_] on likelihood of MI and ischaemic heart disease	Brøndum‐Jacobsen *et al*. (2012)
		Reviews	Beveridge & Witham (2013); Kienreich *et al*. (2013); Norman & Powell (2014)
Stroke		Review	Makariou *et al*. (2014)
		77% of patients had insufficient vitamin D levels	Poole *et al*. (2006)
		OR = 1.52 for ‘low’ *versus* ‘high’ [25(OH)D_3_]	Sun *et al*. (2012)
		OR = 1.33–1.85 for ‘low’ *versus* ‘high’ [25(OH)D_3_]	Judd *et al*. (2016)
		Poor 90‐day outcome and larger infarct volume strongly related to lower vitamin D levels	Turetsky *et al*. (2015)
	Ischaemic only (no effect on haemorrhagic) possibly implying a role in clotting	Strong inverse relation with [25(OH)D_3_]	Brøndum‐Jacobsen *et al*. (2013)
	Ischaemic	[25(OH)D_3_] a very good predictor of favourable outcomes (OR = 1.9)	Park *et al*. (2015)
		OR = 1.6 or more for low *versus* high [25(OH)D_3_]	Chaudhuri *et al*. (2014)
Venous thromboembolism		1.37 RR lowest to highest tertile for seasonally adjusted [25(OH)D_3_]	Brøndum‐Jacobsen *et al*. (2013)
**Metabolic**			
Obesity		Obesity negatively correlated with serum [25(OH)D_3_]	Jamal‐Allial *et al*. (2014)
Type 2 diabetes (T2D)		Hazard ratio (HR) = 1.45 for bottom *versus* top quartile of [25(OH)D_3_] (and also raised ferritin levels in disease cohort; Forouhi *et al*., 2007)	Forouhi *et al*. (2012)
		1.5 HR for bottom *versus* top quartile of [25(OH)D_3_]	Afzal *et al*. (2013)
		1.25 RR for a reduction of [25(OH)D_3_] by 25 nM, but associative and not causative	Ye *et al*. (2015)
		Relationship with body mass index (BMI) and T2D susceptibility mediated *via* low vitamin D levels	Afzal *et al*. (2014c)
**Neurodegenerative and related**			
Amyotrophic lateral sclerosis		No benefits from vitamin D supplements	Karam *et al*. (2013)
Alzheimer's		OR = 0.23 for highest *versus* lowest quintile of vitamin D intake	Annweiler *et al*. (2012)
		HR = 2.25 for [25(OH)D_3_] < 25 nM and 1.53 for 25–50 nM	Littlejohns *et al*. (2014)
		Meta‐analysis: 21% increased risk for [25(OH)D_3_] < 50 nM	Shen & Ji (2015)
		Meta‐analyses	Banerjee *et al*. (2015); Lu'o'ng & Nguyên (2013)
		HR = 1.25 if [25(OH)D_3_] < 25 nM	Afzal *et al*. (2014)
Cognition		Meta‐analysis	van der Schaft *et al*. (2013)
		Rates of decline in episodic memory and executive function greater in vitamin D deficiency	Miller *et al*. (2015)
		Poorer cognitive performance if vitamin D < 10 ng ml^−1^ (Framingham heart study)	Karakis *et al*. (2016)
		Cognitive scores in Minimental State Examination (MMSE) correlated with vitamin D levels	Peterson *et al*. (2012)
Huntington's		89% of patients ‘deficient’ in vitamin D. Positive association between serum [25(OH)D_3_] levels and functional ambulation classification (FAC) scores	Chel *et al*. (2013)
Myalgic encephalomyelitis/ chronic fatigue syndrome			Berkovitz *et al*. (2009); Witham *et al*. (2014)
Parkinson's		OR = 2.2 for [25(OH)D_3_] < 50 nM	Lv *et al*. (2014)
		Correlation of vitamin D levels with improved cognition and mood	Peterson *et al*. (2013)
		Meta‐analysis	Zhao *et al*. (2013)

### (1) Evidence that a low 25(OH)D_3_ level is an effect rather than a cause of inflammation

**Figure 2:**
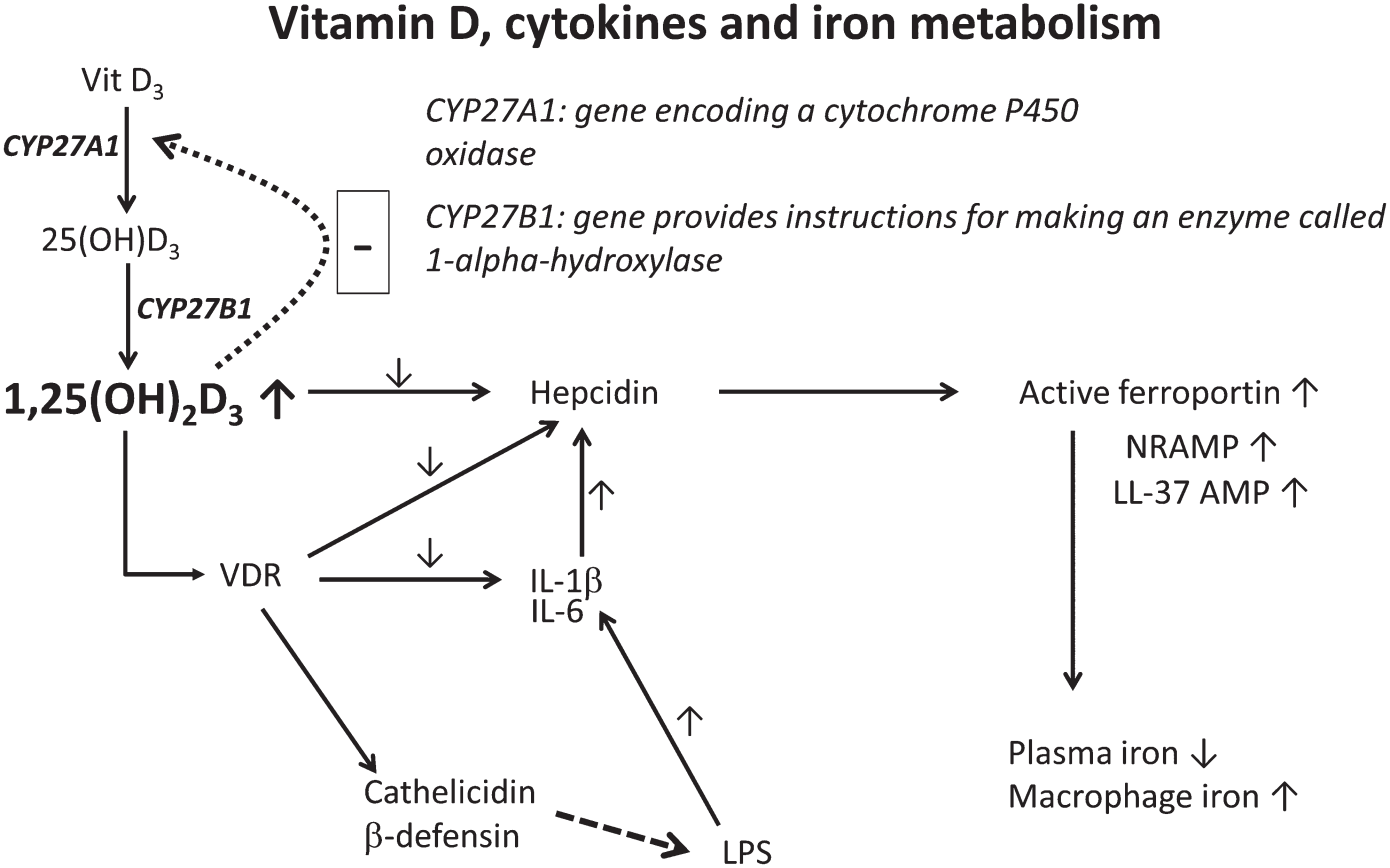
A simplified scheme showing the links between Vitamin D, cytokines and iron metabolism during chronic inflammation. 25(OH)D_3_, 25‐hydroxyvitamin D; 1,25(OH)2D_3_, calcitriol or 1,25‐dihydroxycholecalciferol; IL, interleukin; LL‐37AMP, antimicrobial peptide LL‐37; LPS, lipopolysaccharide; NRAMP, natural resistance‐associated macrophage proteins; VDR, vitamin D receptor.

Inflammatory cytokines can induce expression of both the vitamin D receptor (VDR) and the cytochrome P450 enzyme CYP27B1 that converts 25(OH)D_3_ to 1,25‐dihydroxyvitamin D_3_ (1,25(OH)_2_D_3_); 1,25(OH)_2_D_3_ suppresses elements of the adaptive immune system while stimulating elements of the innate immune system (Bikle, 2009). In addition (Bell, Shaw & Turner, 1984) 1,25(OH)_2_D_3_ inhibits hepatic production of 25(OH)D_3_, explaining how inflammation can simultaneously cause high 1,25(OH)_2_D_3_ and low 25(OH)D_3_ levels (Fig. 2). Obviously measuring 25(OH)D_3_ levels alone will be a rather poor guide to the effective vitamin D status.

Mangin *et al*. (2014) and Waldron *et al*. (2013) therefore suggested that low 25(OH)D_3_ concentration is a consequence of chronic inflammation rather than a cause, and that tissue bacteria could be responsible for an inflammatory disease process resulting in high 1,25(OH)_2_D_3_ and low 25(OH)D_3_ levels (see also Waterhouse, Perez & Albert, 2009).

One signalling role of 1,25(OH)_2_D_3_ is to activate the VDR (Carlberg & Campbell, 2013; Kongsbak *et al*., 2013; Schauber *et al*., 2007), a transcription factor that can induce the expression of over 900 genes. From an infection or innate immunity perspective, it is important that the products of these genes include antimicrobial peptides (AMPs) (Bartley, 2010a; Coussens, Martineau & Wilkinson, 2014; Fabri *et al*., 2011; Liu *et al*., 2006; Proal *et al*., 2014; Youssef *et al*., 2011) such as cathelicidin and beta defensins (Fig. 2) which are known to attack pathogens (Nnoaham & Clarke, 2008).

There is now a complex (Nama *et al*., 2016) and often contradictory literature (Kearns *et al*., 2015) regarding vitamin D supplementation. Some studies have highlighted a relationship between low 25(OH)D_3_ levels and Alzheimer's disease (Banerjee *et al*., 2015; Littlejohns *et al*., 2014; Lu'o'ng & Nguyên, 2013; Miller *et al*., 2015; Shen & Ji, 2015) (see also Table 1). A naïve view [recapitulating the now‐discredited ‘crossover theorem’ (Chance & Williams, 1955)] would suggest that vitamin D supplementation could be a solution. To date, however, there is little evidence for clinical benefits from vitamin D (Bjelakovic *et al*., 2014a,*b*; Brøndum‐Jacobsen *et al*., 2015; Karam *et al*., 2013; Makariou *et al*., 2014; Newberry *et al*., 2014; Pilz *et al*., 2015; Witham *et al*., 2015). This may reflect different populations of individuals who respond differently to vitamin D_3_ supplementation (Carlberg *et al*., 2013; Ryynänen *et al*., 2014; Saksa *et al*., 2015), or perhaps the simultaneous presence of individuals in which the VDR responds to vitamin D as an agonist or an antagonist (Anami *et al*., 2014). It is known that small changes in the sequence of the VDR can have major phenotypic effects, e.g. an odds ratio (OR) for stroke of 2.97 was calculated for one particular allele (Prabhakar *et al*., 2015). A systems biologist will recognise that supplementation may not be the answer, and indeed there is some evidence for the opposite effect (Mangin *et al*., 2014; Marshall, 2008; Proal *et al*., 2015). Clearly we need to clarify the different roles of 25(OH)D_3_ and 1,25(OH)_2_D_3_, and any effects of chronic conditions on the CYP enzymes that produce them. Biomarkers [such as taurinuria (Chesney, Dabbagh & Han, 2015) for genuine vitamin D deficiency may prove useful in this work.

Finally, we recognise that signalling can be effected both by changes in the amplitude of a signal and also by changes in its frequency, as is the case for the apoptotic *versus* proliferative effects of nuclear factor‐kappa B (NF‐κB) (Ashall *et al*., 2009; Kell, 2006; Nelson *et al*., 2004). Vitamin D is known to have significant effects on NF‐κB (Chen *et al*., 2013; Szeto *et al*., 2007; Wu *et al*., 2010,*b*) and VDR expression levels are partly dependent on extracellular signal‐related kinase (ERK) (Ordóñez‐Morán & Muñoz, 2009), which also oscillates (Waters *et al*., 2014). Vitamin D_3_ also regulates circadian genes (Gutierrez‐Monreal *et al*., 2014). Consequently, ‘oscillation‐based’ explanations of signal transduction may be relevant to the role of vitamin D in inflammation.

It is thus clear (e.g. Bartley, 2010a, *b*; Mangin *et al*., 2014)) that there are major interactions between inflammation, infection, and vitamin D metabolism [including elements of iron and vitamin D metabolism (Zughaier *et al*., 2014), see below].

### (2) Vitamin D and iron metabolism mediated by hepcidin

The protein hepcidin is a key regulator of mammalian iron metabolism (Ganz, 2006; Ganz & Nemeth, 2012; Michels *et al*., 2015; Reichert *et al*., 2017; Vyoral & Jiri, 2017; Zaritsky *et al*., 2009). As Zughaier *et al*. (2014) comment, 25(OH)D_3_ concentrations (as modified via the addition of 1,25(OH)2D but assessed by serum 25‐hydroxyvitamin D (25(OH)D)) are inversely associated with hepcidin concentrations and are positively associated with levels of haemoglobin and iron' (Carvalho *et al*., 2011; Icardi *et al*., 2013; Perlstein *et al*., 2011; Zaritsky *et al*., 2009), while hepcidin and 1,25(OH)_2_D_3_ stimulated a strong increase in levels of ferroportin 1, natural resistance associated macrophage protein 1 (NRAMP1) and LL‐37 antimicrobial peptide, which lead to a reduction in plasma iron levels (Fig. 2). The inflammatory cytokine interleukin‐6 (IL‐6) also induces hepcidin production (Chesney *et al*., 2015; Ganz & Nemeth, 2015; Lee *et al*., 2005; Nemeth *et al*., 2004).

Zughaier *et al*. (2014, p. e23) noted that ‘LPS is a major component of microbial translocation seen during chronic inflammation (Layoun & Santos, 2012; Theurl *et al*., 2008; Wang *et al*., 2009). LPS induces both hepcidin and IL‐6 expression whereas LL‐37 binds and neutralizes LPS activity (Zughaier, Shafer & Stephens, 2005)’. Increases in 1,25(OH)_2_D_3_ cause hepcidin levels to decrease, *via* binding of the VDR to hepcidin's promoter (Bacchetta *et al*., 2014,*b*), and levels of IL‐1β and IL‐6 are also decreased (Fig. 2) [exacerbating the decrease in hepcidin (Ganz & Nemeth, 2015)]. Decreased hepcidin levels enhance the surface exposure of ferroportin, while associated increases in NRAMP and LL‐37 lead to potential hyperferraemia (Fig. 2). The increase in hepcidin levels *via* IL‐6 (Layoun & Santos, 2012; Wang *et al*., 2009) is partly mediated by microRNA‐155 (mi‐RNA‐155) that increases with increasing LPS levels and is inversely related to vitamin D levels (Li *et al*., 2014). Thus, while the process is complex, it does appear that vitamin D metabolism is intimately involved in the microbial processes that could lead to chronic, inflammatory disease.

## V. STEP 0: IRON DYSREGULATION CAUSED BY EXTERNALLY INDUCED STRESSES

As any student of metabolic control analysis (Fell, 1996; Fell & Thomas, 1995; Heinrich & Rapoport, 1974; Kacser & Burns, 1973) or systems biology knows, individual metabolic steps alone rarely control the flux in biochemical networks. Thus, although we attempt to order the steps in Fig. 1A temporally, it is hard to be certain about the exact sequence of causality. Nonetheless, iron dysregulation is step 0 in our systems biology approach because of two outcomes: (*i*) the production of hydroxyl radicals, catalysed by ‘free’ iron that can itself lead to cell death (step 1); and (*ii*) the iron‐based reactivation of dormant microbes (step 2). In this section we concentrate on the first mechanism. Many reviews of general iron metabolism are available elsewhere (Kell, 2009, 2010; Kell *et al*., 2015; Kell & Pretorius, 2014, 2015b; Chifman *et al*., 2012; Mitchell & Mendes, 2013; Parmar *et al*., 2017).

Iron can have negative effects, as reviewed extensively elsewhere (e.g. Altamura & Muckenthaler, 2009; Anderson & Wang, 2012; Berg & Youdim, 2006; Bush & Tanzi, 2008; Castellani *et al*., 2012; Chifman, Laubenbacher & Torti, 2014; Collingwood & Davidson, 2014; Crichton, 2016; Crichton, Dexter & Ward, 2011; Dixon & Stockwell, 2013; Farina *et al*., 2013; Ganz & Nemeth, 2015; Hansen, Moen & Mandrup‐Poulsen, 2014; Jellen, Beard & Jones, 2009; Kell, 2009, 2010; Kell & Pretorius, 2014; Koskenkorva‐Frank *et al*., 2013; Lehmann *et al*., 2015; Levi & Finazzi, 2014; Mollet *et al*., 2016; Muhoberac & Vidal, 2013; Muller & Leavitt, 2014; Nikonorov *et al*., 2015; Núñez *et al*., 2012; Oliveira, Rocha & Fernandes, 2014; Peters, Connor & Meadowcroft, 2015; Pisano, Lombardi & Fracanzani, 2016; Rouault, 2016; Schneider, 2016; Shovlin *et al*., 2015, 2016; Simcox & McClain, 2013; Stankiewicz, Neema & Ceccarelli, 2014; Stephenson *et al*., 2014; Sullivan, 2009; Thuret, 2013; Vinchi *et al*., 2014; Weinreb *et al*., 2013; Yin *et al*., 2012; Zhao *et al*., 2012; Zhuang, Han & Yang, 2014), as well as being an essential nutrient for cell growth (*cf*. Posey & Gherardini, 2000). ‘Iron’ can be present as Fe^2+^ and Fe^3+^ valencies, and also has six liganding sites (four ‘equatorial’, two ‘polar’) that affect its reactivity in two linked reactions involving peroxide and superoxide (molecules that are always present in aerobic systems). The amount of ‘free’ iron varies, but Fe(III) salts are virtually insoluble at neutral pH (explaining the need for microbial siderophores, see Sections V and VII); the typical cytoplasmic levels of ‘free’ iron are in the range 1–10 µM (Hider & Kong, 2013).

Both hydrogen peroxide and superoxide are common products of the partial reduction of oxygen by mitochondria, among other sources (Kell, 2009). Hydrogen peroxide can react with free or poorly liganded Fe(II) in the Fenton reaction (Wardman & Candeias, 1996), leading to the production of very reactive and damaging hydroxyl radicals (OH^•^).

(1) $$\mathrm{Fe}\left(\mathrm{II}\right)+H_{2}O_{2}\to \mathrm{Fe}\left(\mathrm{III}\right)+{\mathrm{OH}}^{-}+{\mathrm{OH}}^{•}$$

The ferric iron can then react with superoxide in the Haber–Weiss reaction (Kehrer, 2000) generating Fe(II) again, thereby effecting redox cycling:

(2) $${O_{2}}^{•-}+\mathrm{Fe}\left(\mathrm{III}\right)\to O_{2}+\mathrm{Fe}\left(\mathrm{II}\right)$$

In other words, catalytic quantities of unliganded or poorly liganded iron can lead to a continuing flux of hydroxyl radicals. These react in nanoseconds with almost anything, and their existence can be detected *via* the products of such reactions, including 8‐hydroxy‐guanine (Shin *et al*., 2001), 8‐hydroxy‐2′‐deoxy‐guanosine (Loft *et al*., 1993; Migliore *et al*., 2005), 4‐hydroxy‐nonenal (Ayala, Muñoz & Argüelles, 2014; Petersen & Doorn, 2004; Tsikas, 2017), various isoprostanes (Davì, Falco & Patrono, 2004; Montuschi, Barnes & Roberts II, 2007, 2004; Montuschi *et al*., 1998, 2000; Morrow, 2005; Schwedhelm & Boger, 2003) and malondialdehyde (Ayala *et al*., 2014; Del Rio, Stewart & Pellegrini, 2005; Janero, 1990; Tsikas, 2017).

This iron dysregulation can be initiated by a multitude of factors that cause cell death, which will release free iron into the bloodstream, whence it can be disseminated throughout the body (Kell & Pretorius, 2014). Such factors include mechanical damage [including trauma (Gorbunov *et al*., 2006, 2005, 2003; Zhang *et al*., 2013) and dysbiosis], nutritional stress (Schaffer, 2003, 2016), pharmacological stress (Pirmohamed *et al*., 2004), oxidative stress (Crichton, 2016; Kerley *et al*., 2018) and others (Nanba *et al*., 2016), many of which also involve the production of stress hormones.

## VI. STEP 1: IRON DYSREGULATION LEADING TO CELL DEATH

Fenton reactions within the cell will potentially result in death *via* apoptosis (Lee *et al*., 2006; Li *et al*., 2016), ferroptosis (Dixon *et al*., 2012; Dong *et al*., 2015; Imai *et al*., 2017; Yang & Stockwell, 2016; Yu *et al*., 2017), and necrosis (Dong *et al*., 2015; Traoré & Meyer, 2007). These processes have been reviewed previously (Kell, 2009, 2010; Kell & Pretorius, 2014), but we here draw attention to the following: (*i*) the reducing agent ascorbic acid (vitamin C) actually becomes a pro‐oxidant when poorly liganded, e.g. with ligands such as ethylene diamine tetraacetate (EDTA) (Kell, 2009); and (*ii*) ferritin is an intracellular marker, so that the serum ferritin level (widely but erroneously used as a measure of iron status) is simply a sign of cell death (Kell & Pretorius, 2014). Indeed, cell death can be autocatalytic, as serum ferritin can lose its iron component (Arosio, Yokota & Drysdale, 1977; Konz *et al*., 2013; Nielsen *et al*., 2000; Watanabe *et al*., 2001; Yamanishi *et al*., 2002), such that cell death liberates free iron that, *via* further Fenton and Haber–Weiss reactions, can cause further cell death.

**Figure 3:**
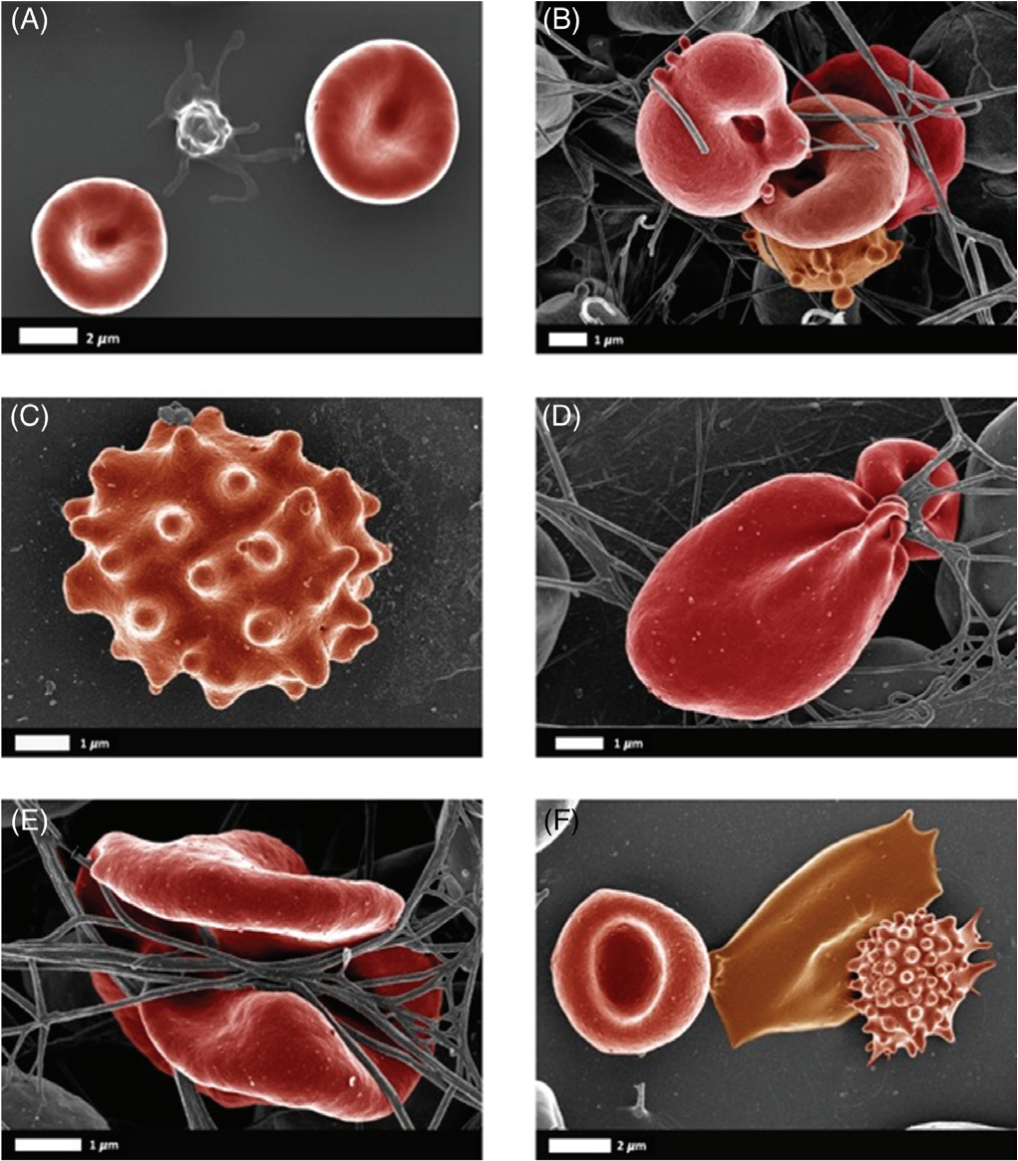
Examples of eryptotic red blood cells (RBCs) in inflammation. (A) Healthy RBCs with a platelet; (B) Type 2 diabetes (Pretorius et al., 2015); (C, D) Parkinson's disease (Pretorius et al., 2014b); (E) Rheumatoid arthritis (Olumuyiwa‐Akeredolu et al., 2017); (F) healthy whole blood exposed to interleukin‐8 (Bester & Pretorius, 2016).

In contrast to apoptosis in nucleated cells, programmed cell death in red blood cells (RBCs) is known as eryptosis (Bissinger *et al*., 2013; Föller *et al*., 2008; Lang & Lang, 2015; E. Lang, Qadri & Lang, 2012a; Lang *et al*., 2010; F. Lang, Lang & Foller, 2012b; Lang & Qadri, 2012; Pretorius, du Plooy & Bester, 2016b; Qadri *et al*., 2011; Qadri *et al*., 2016; Qadri *et al*., 2012). It causes the release of haem from RBCs, which can eventually lead to the presence of free ‘iron’. The physiological processes taking place during eryptosis are similar to those of apoptosis, but without the involvement of the nucleus and mitochondria. Examples of eryptotic RBCs in the presence of inflammation are shown in Fig. 3A–E; Fig. 3F is an example of eryptosis induced by addition of IL‐8 to healthy whole blood.

## VII. STEP 2: MICROBIAL REACTIVATION AND VIRULENCE VIA FREE ‘IRON’

‘Chocolate’ agar is a medium widely used for assaying bacteria *via* their growth, and is essentially heated blood. However, bacteria proliferate much less well in actual blood, partly due to the presence of antimicrobial components and the innate immune system but also because healthy blood *in vivo* normally has almost no free iron available (1–10 µM) (Armitage & Drakesmith, 2014; Chu *et al*., 2010; Haley & Skaar, 2012; Sivick & Mobley, 2010; Subashchandrabose & Mobley, 2015; Wessling‐Resnick, 2010). Indeed iron‐withholding (Ganz, 2009; Jurado, 1997; Nevitt, 2011; Weinberg, 2009; Weinberg & Miklossy, 2008) is a major strategy used by hosts to inhibit the growth of microbial invaders. This is often described as a ‘battle’ (Armitage & Drakesmith, 2014; Carver, 2018; Chu *et al*., 2010; Damron *et al*., 2016; Fischbach *et al*., 2006; Haley & Skaar, 2012; Pich & Merrell, 2013; Skaar, 2010; Stijlemans *et al*., 2015) or ‘struggle’ (Markel *et al*., 2007; Nairz *et al*., 2010; Reid, Anderson & Lamont, 2009) for iron between the host and invader.

In consequence, the likelihood of infection is greatly enhanced when free iron levels are raised (Boyanova, 2011; Braun, 2005; Eichhorn *et al*., 2006; Ishida & Johansen, 2014; Mittal *et al*., 2008; Nevitt, 2011; Ngok‐Ngam *et al*., 2009; Rodriguez & Smith, 2003; Sritharan, 2006; Su *et al*., 2009; Sutak *et al*., 2008; Vasil & Ochsner, 1999), and indeed the ‘virulence’ of microbes is strongly correlated with their expression of siderophore (iron‐binding) or iron transporter (Do, Zafar & Saier Jr, 2017; Tang & Saier Jr, 2014) genes. In addition, siderophores can act directly to induce cytokine expression (Holden *et al*., 2016).

An obvious corollary is that iron‐overload disorders such as hereditary haemochromatosis or the thalassaemias will result in a significantly higher susceptibility to infection (Ashrafian, 2003; Barton & Acton, 2009; Christopher, 1985; Khan, Fisher & Khakoo, 2007; Moalem, Weinberg & Percy, 2004; Muench, 1989; Weinberg, 1978, 2009). We suggest herein that it is a combination of free iron and microbial reactivation that is key to understanding chronic, inflammatory disease.

## VIII. STEP 3: IRON DYSREGULATION AND DISEASE

Although we suspect that the greater significance of free iron in chronic, inflammatory diseases is *via* microbial activation (Fig. 1) rather than *via* the Fenton and Haber–Weiss reactions and oxidative stress, there is no doubt that excess iron is itself directly involved in a variety of diseases (Table 2).

**Table 2:** Selected diseases in which iron dysregulation takes place

Disease	Comments	Selected references
Alzheimer's disease	Likely role of iron binding to amyloid proteins	Altamura & Muckenthaler (2009); Ayton *et al*. (2015, 2017); Barnham & Bush (2008); Belaidi & Bush (2016); Casadesus *et al*. (2004); Castellani *et al*. (2012); Crichton (2016); Crichton *et al*. (2011); Gallagher *et al*. (2012); Gargano & Hughes (2014); Grünblatt *et al*. (2011); Peters *et al*. (2015); Pretorius *et al*. (2016a); Sternberg *et al*. (2017); Telling *et al*. (2017); van Duijn *et al*. (2017); Wood (2015)
Amyotrophic lateral sclerosis (Lou Gehrig's disease)		Hadzhieva *et al*. (2013); Ignjatović *et al*. (2012, 2013); Molfino *et al*. (2009); Oshiro *et al*. (2011); Sheelakumari *et al*. (2016); Wang *et al*. (2011)
Atherosclerosis	Huge levels of iron in atherosclerotic plaques	Altamura & Muckenthaler (2009); Galesloot *et al*. (2015); Kraml (2017); Sharkey‐Toppen *et al*. (2014); Stadler *et al*. (2004); Stanley *et al*. (2006); Sullivan (2009); Winner III *et al*. (2015)
Type 2 diabetes,	Abundant epidemiological evidence	Altamura *et al*. (2017); Ambachew & Biadgo, 2017; Basuli *et al*. (2014); Fernández‐Cao *et al*., 2017; Fernández‐Real *et al*. (2002, 2015); Hansen *et al*. (2014); Huth *et al*. (2015); Kundu *et al*. (2013); Mascitelli *et al*. (2009); Montonen *et al*. (2012); Podmore *et al*. (2016); Simcox & McClain (2013); X. Wang *et al*. (2015b); Zhao *et al*. (2012)
Friedreich's ataxia	Clear mechanistic linkage *via* frataxin, an Fe‐S protein chaperone	(Anzovino *et al*. (2014); Chiang *et al*. (2016); Harding *et al*. (2016); Martelli & Puccio (2014); Richardson *et al*. (2010); Vaubel & Isaya (2013); Wilson (2006)
Oxidative DNA damage	Products of Fenton reaction	Hori *et al*. (2010); Mollet *et al*. (2016); Shaw *et al*. (2017); Singh & Chadha (2016); Zein *et al*. (2017)
Parkinson's disease	Dopamine makes substantia nigra especially sensitive; among the syndromes with the most evidence for iron involvement	Altamura & Muckenthaler (2009); Barnham & Bush (2008); Berg (2007); Brar *et al*. (2009); Costa‐Mallen *et al*. (2017); Crichton *et al*. (2011); Dusek *et al*. (2014); Hare *et al*. (2014); Lee & Andersen (2010); Maes *et al*. (2017); Mochizuki & Yasuda (2012); Weinreb *et al*. (2013)
Pre‐eclampsia	Considerable evidence of iron dysregulation	Entman *et al*. (1987); Kell (2009); Kell & Kenny (2016); Kenny & Kell (2018); Kerley *et al*. (2018); Rayman *et al*. (2002); Serdar *et al*. (2006); Toldi *et al*. (2010)
Rheumatoid arthritis	Considerable evidence of iron dysregulation	Baker & Ghio (2009); Dombrecht *et al*. (2004); Donnelly *et al*. (2010); Stefanova *et al*. (2016)
Stroke	Considerable evidence of iron dysregulation	Armengou & Davalos (2002); Petrova *et al*. (2016); Selim & Ratan (2004); Tuo *et al*. (2017)

## IX. STEP 4: MICROBES CAN PRODUCE AND SHED INFLAMMAGENS SUCH AS LPS AND LTA

The cell walls of Gram‐negative and Gram‐positive bacteria contain significant amounts of LPS and LTA that can become detached in response to different environmental and physiological signals (e.g. Watson *et al*., 1977). When shed into the host, LPS is known as endotoxin. The most extreme example of microbial shedding of inflammatory material of this type is in a condition known as the Jarisch–Herxheimer reaction (Almeida, Estanqueiro & Salgado, 2016; Belum *et al*., 2013; Cheung & Chee, 2009; Guerrier & D'Ortenzio, 2013; Kadam *et al*., 2015; Pound & May, 2005; See, Scott & Levin, 2005), which is essentially an uncontrolled cytokine storm (see Section X) caused by the rapid release of inflammagenic cell wall materials from microbes, often following bactericidal antibiotic treatment (Lepper *et al*., 2002).

### (1) LPS as the inflammagen par excellence

The inflammagenic potency of LPS is so great that it is commonly (and ironically) even used as a model to induce symptoms more or less similar to many of the inflammatory diseases of interest. Typically this involves injecting LPS at the site of interest for such diseases. Examples of the use of endotoxin in this way include pre‐eclampsia (Cotechini *et al*., 2014; Faas *et al*., 1994; Faas *et al*., 2000; Lin *et al*., 2012; Liu *et al*., 2017; Rademacher, Gumaa & Scioscia, 2007; Sakawi *et al*., 2000; Williamson *et al*., 2016; Xue *et al*., 2015), Alzheimer's (Zhan *et al*., 2015, 2016), Parkinson's (Barnum & Tansey, 2010; Byler *et al*., 2009; Cunningham *et al*., 2005; He *et al*., 2013; Hoban *et al*., 2013; Hritcu & Ciobica, 2013; Hritcu *et al*., 2011; Liu & Bing, 2011; Miller *et al*., 2009; Orr, Rowe & Halliday, 2002; Santiago *et al*., 2010; Tufekci, Genc & Genc, 2011; Z. Zhang *et al*., 2012), rheumatoid arthritis (Izui, Eisenberg & Dixon, 1979; Nemeth *et al*., 1985), atherosclerosis (Khedoe *et al*., 2013), multiple sclerosis (di Penta *et al*., 2013; Nguyen *et al*., 2004), Guillain‐Barré syndrome (Prendergast & Moran, 2000), sepsis (Lewis, Seymour & Rosengart, 2016; Remick & Ward, 2005), and stroke (Becker *et al*., 2005; Doll *et al*., 2015; Shim & Wong, 2016). This far‐from‐exhaustive list illustrates well the generality of this phenomenon. In cases of stroke, infection is very common, and leads to a worse prognosis; in some cases antibiotics worsen it further (Becker *et al*., 2016), consistent with the view that the infecting organisms were already present, and that there is an active role of LPS shedding. We note too that some molecules such as P‐type inositol phosphate glycans can act as LPS mimics (Robillard *et al*., 2016). This is especially well established in pre‐eclampsia (e.g. Dawonauth *et al*., 2014; Kenny & Kell, 2018; Robillard *et al*., 2016; Scioscia *et al*., 2012, 2011; Williams *et al*., 2007) but seems to have been little investigated elsewhere.

Consistent with the above (and see de Punder & Pruimboom, 2015; Kell & Pretorius, 2015a), Table 3 lists a variety of ‘natural’ (i.e. non‐experimental) chronic inflammatory diseases for which it has been shown that steady‐state endotoxin (LPS) levels are raised and Table 4 presents examples of diseases in which raised levels of lipopolysaccharide binding protein (LBP) have been observed.

**Table 3:** Diseases in which levels of lipopolysaccharide (LPS; endotoxin) are higher in patients than in matched controls

Disease	Comments	Selected references
Alzheimer's disease	At sites of central nervous system (CNS) lesions	Bester *et al*. (2015); Poole *et al*. (2013); Zhan *et al*. (2016)
Amyotrophic lateral sclerosis		Zhang *et al*. (2009)
Atherosclerosis		Kiechl *et al*. (2001); Ostos *et al*. (2002); Stoll *et al*. (2004)
Cancer	Tumours contained high levels of bacteria and LPS	Cummins & Tangney (2013); Geller *et al*. (2017)
Type 2 diabetes	Also bound up with amylin	Andreasen *et al*. (2010); Cani *et al*. (2012); Chen *et al*. (2016); de Kort *et al*. (2011); Jayashree *et al*. (2014); Miklossy *et al*. (2008); Pussinen *et al*. (2011); Vergès *et al*. (2014)
Multiple sclerosis		Ballerini *et al*. (2017); Escribano *et al*. (2017)
Oxidative damage		Duvigneau *et al*. (2008); Escribano *et al*. (2017); Li *et al*. (2016); Ozdemir *et al*. (2007); Ritter *et al*. (2006)
Parkinson's disease		Chang & Li (2011); Chen *et al*. (2018); Forsyth *et al*. (2011); Girard‐Joyal & Ismail (2017); Harris *et al*. (2012); He *et al*. (2013); Hoban *et al*. (2013); Kelly *et al*. (2014); Kim *et al*. (2016)

**Table 4:** Examples of diseases in which raised lipopolysaccharide binding protein (LBP) levels have been observed

Disease	Comments	Selected references
Atherosclerosis		Lepper *et al*. (2011, 2007); Serrano *et al*. (2013); see also Sallam *et al*. (2014)
Type 2 diabetes	High‐fat diet induction and correlation with obesity	Ghanim *et al*. (2009); Moreno‐Navarrete *et al*. (2013); Sakura *et al*. (2017); Sun *et al*. (2010); Tuomi & Logomarsino (2016)
Multiple sclerosis		Escribano *et al*. (2017)
Parkinson's disease		Forsyth *et al*. (2011); Pal *et al*. (2015)
Rheumatoid arthritis		Kim *et al*. (2018); Wen *et al*. (2018)

### (2) LTA shedding from Gram‐positive bacteria

Gram‐positive bacteria have a cell wall structure that differs from that of Gram‐negatives both in its number of barriers and in the fact that the cell wall component equivalent to LPS is lipoteichoic acid (LTA). LTA is equivalently capable of producing an inflammatory response. In contrast to LPS, which mainly interacts with toll‐like receptor 4 (TLR4) (Balasubbramanian *et al*., 2017; Hoshino *et al*., 1999; Kell & Pretorius, 2015a; Lien *et al*., 2000; Poltorak *et al*., 1998), LTA stimulates target cells mainly by activating toll‐like receptor 2 (TLR2) (Ishii & Akira, 2004; Jiménez‐Dalmaroni, Gerswhin & Adamopoulos, 2016; Kawai & Akira, 2011; Kumar, Kawai & Akira, 2011; Kumar *et al*., 2013; Y. Liu *et al*., 2014; Mukherjee, Karmakar & Babu, 2016; Oliveira‐Nascimento, Massari & Wetzler, 2012; Schwandner *et al*., 1999; Underhill *et al*., 1999; Zähringer *et al*., 2008). The glycolipid anchor of LTA plays a central role, analogous to lipid A of LPS (Morath, von Aulock & Hartung, 2005).

LTA species have been rather less studied from the point of view of inflammagenesis than have LPS forms, but they clearly reside in the blood and are inflammagens (Barbero‐Becerra *et al*., 2011; Cinar *et al*., 2013; Hoogerwerf *et al*., 2009; Levels *et al*., 2003; Pirillo, Catapano & Norata, 2015). In some respects (see Section X), LTAs may be even more potent than LPS species (Pretorius *et al*., 2018a).

## X. STEP 5: INDUCTION BY LPS AND LTA OF INFLAMMATORY CYTOKINES

**Figure 4:**
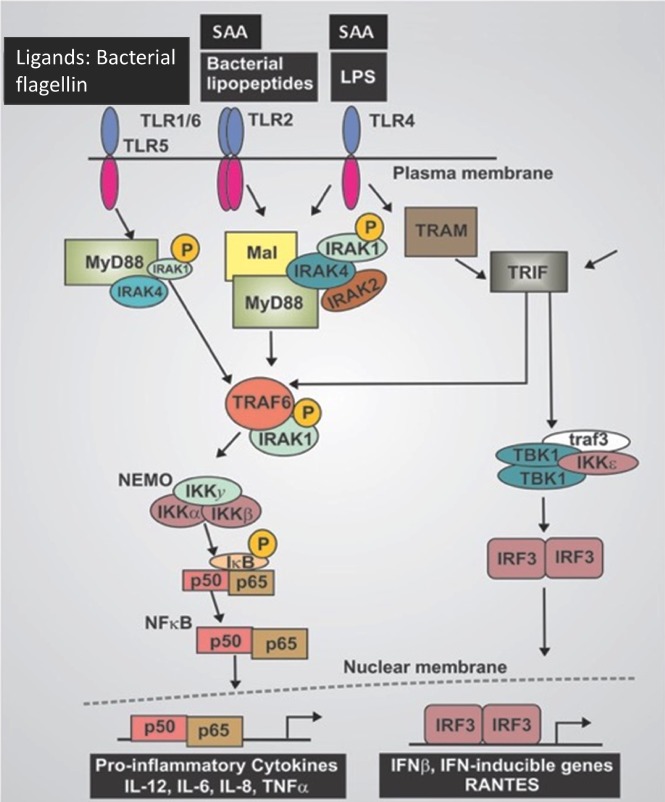
Lipopolysaccharide (LPS)‐ and serum amyloid A (SAA)‐mediated cellular production of inflammatory cytokines. Canonical pathway of LPS‐mediated release and nuclear translocation of nuclear factor‐kappa B (NF‐ κB) (based on O'Neill et al., 2009). IKK, IκB kinase complex; INF, interferon; IRF3, interferon regulatory factor 3; MyD88, myeloid differentiation primary response 88; NEMO, NF‐κB essential modulator; p50, NF‐κB subunit, p50; p65, transcription factor p65 also known as nuclear factor NF‐kappa‐B p65 subunit; RANTES, hemokine (C‐C motif) ligand 5; SAA, Serum amyloid A; TBK1, TANK‐binding kinase 1; TIRF, TIR‐domain‐containing adapter‐inducing interferon‐β; TLR, Toll‐like receptor; TRAF, TNF receptor associated factor; TRAM, TRIF‐related adaptor molecule.

**Figure 5:**
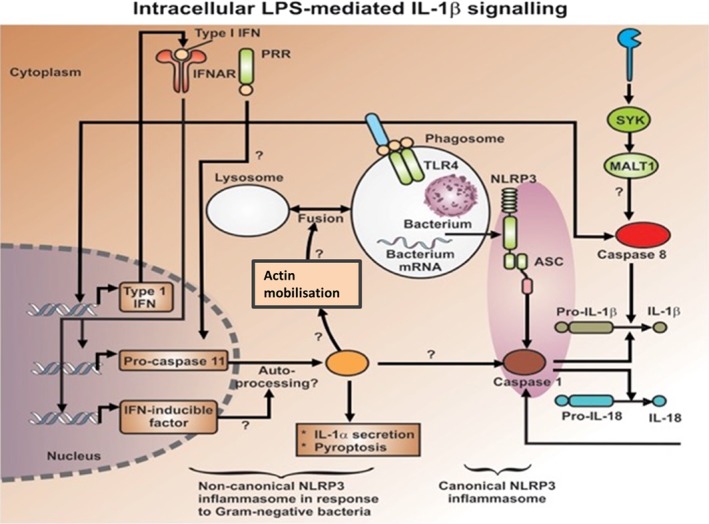
Intracellular lipopolysaccharide (LPS)‐mediated activation of caspase‐1 leading to interleukin 1β (IL‐1β) production (after Latz et al., 2013). ASC, caspase activation and recruitment domain; IL, interleukin; INF, type 1 interferon; INFAR, interferon receptor; MALT1, mucosa‐associated‐lymphoid‐tissue lymphoma‐translocation gene 1; NTLP3, nucleotide‐binding oligomerization domain‐like receptor family, pyrin domain‐containing‐3; PRR, pattern recognition receptor; SYK, spleen tyrosine kinase; TLR4, Toll‐like receptor 4.

The induction of inflammatory cytokines by LPS and LTA has been reviewed numerous times (e.g. (Kell & Pretorius, 2015a, 2016; Latz, Xiao & Stutz, 2013; O'Neill, Bryant & Doyle, 2009). The basic pathways (Latz *et al*., 2013; O'Neill *et al*., 2009) that lead from TLR binding to inflammatory cytokine production are shown in Figs 4 and 5 [reproduced from Kell & Pretorius (2015a) under a CC‐BY license]. They result in increased levels of circulating inflammatory cytokines and other ‘acute phase’ biomarkers, in particular IL‐1β, IL‐6, IL‐8 and tumour necrosis factor α (TNFα) (e.g. Pindjakova *et al*., 2017; van Rijn *et al*., 2016). In some cases (e.g IL‐1β), these can serve as ligands that stimulate their own synthesis (Brown *et al*., 2013; Small *et al*., 2011). A variety of small‐molecule (Donia & Fischbach, 2015) microbial products besides LPS and LTA, such as long‐ (Schirmer *et al*., 2016) and short‐chain (Thorburn, Macia & Mackay, 2014) fatty acids, can also lead to or modulate the formation of inflammatory cytokines. A variety of other molecules are markers of systemic inflammation; these include C‐reactive protein, serum amyloid A and fibrinogen (e.g. Bickel *et al*., 2002; Çetinkaya *et al*., 2009; Davalos & Akassoglou, 2012; De Buck *et al*., 2016; deRosset & Strutz, 2015; Hesselink, Aarden & Swaak, 2003; Kaptoge *et al*., 2012; Ridker & Silvertown, 2008; Song *et al*., 2006; Yildirim, Hur & Kokturk, 2013) – interestingly all correlate inversely with socioeconomic status (Jousilahti *et al*., 2003). The role of ferritin, another ‘acute‐phase protein’ synthesised in response to infection/inflammation, has been discussed in detail elsewhere (Kell & Pretorius, 2014).

## XI. STEP 6: INDUCTION OF FIBRIN AMYLOID FORMATION BY ‘IRON’, LPS AND LTA

**Figure 6:**
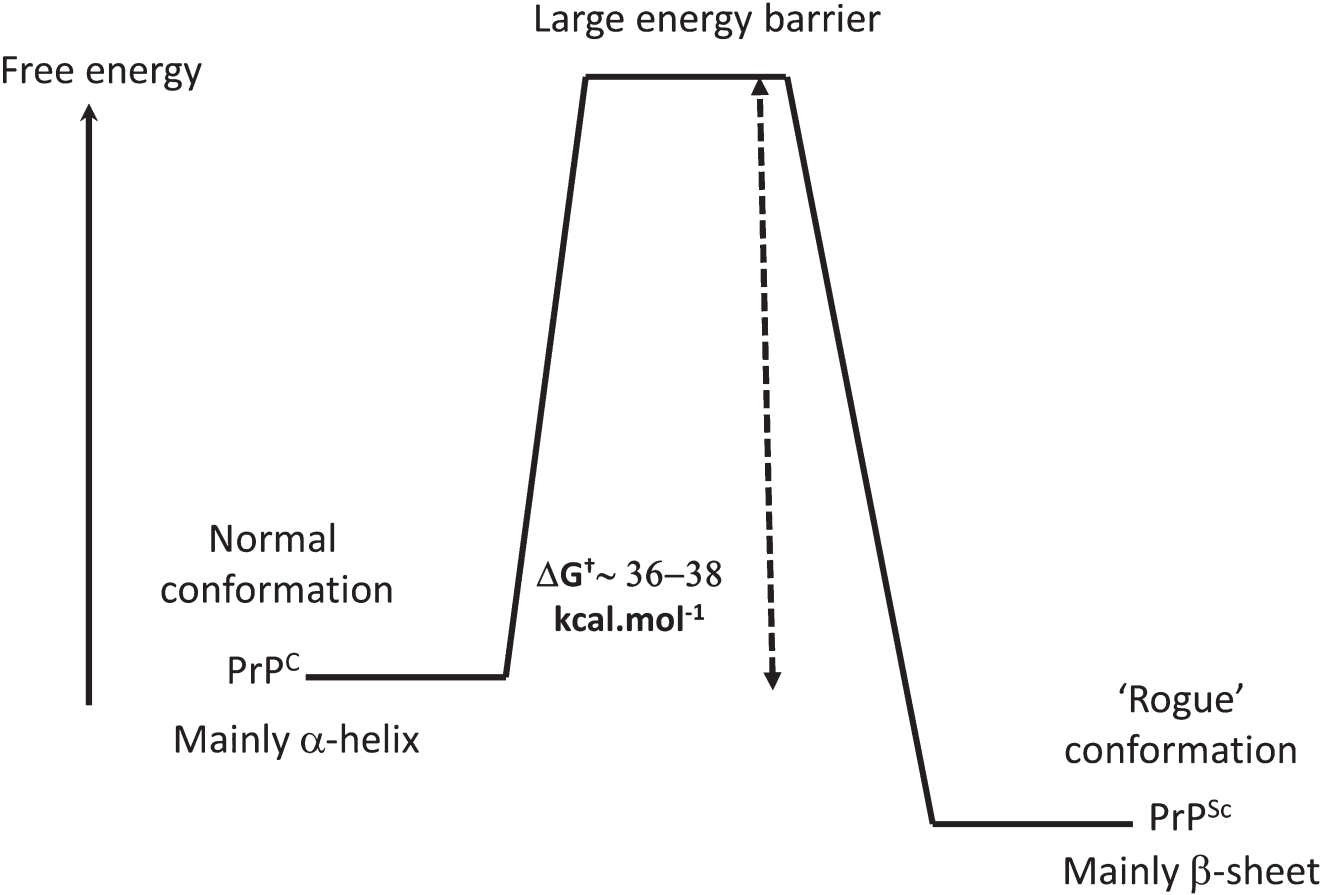
Energy barriers in prion protein formation [based on Cohen & Prusiner (1998) and Kell & Pretorius (2017a)]. Normal cell‐surface glycoprotein: PrP^c^; prion protein scrapie associated: PRP^SC^; ΔG^†^ free energy of activation.

‘Amyloid’, more specifically an amyloid protein fibril, is defined formally (Sipe *et al*., 2014, p. 221) as ‘a protein that is deposited as insoluble fibrils, mainly in the extracellular spaces of organs and tissues as a result of sequential changes in protein folding that result in a condition known as amyloidosis’. As with prions (Aguzzi & Lakkaraju, 2016; Kell & Pretorius, 2017a; Prusiner, 1998; Prusiner *et al*., 2015), there is (or need be) no change in the primary sequence when a normally soluble protein adopts an insoluble amyloid form. Anfinsen's (1973) classical experiments had implied that the primary sequence alone can be sufficient to guide normal folding and that folding was to the state of lowest free energy. The existence of more stable conformations than those first formed upon folding implies, in contrast to this, that there is a large kinetic barrier between the most common conformation and the folded amyloid form(s) of lower free energy (Cohen & Prusiner, 1998) (Fig. 6). As many as 50 ‘amyloid’ diseases are now established (Ankarcrona *et al*., 2016; Buell, Dobson & Knowles, 2014; Dobson, 2013; Hung *et al*., 2016; Ke *et al*., 2017; Kholová & Niessen, 2005; Knowles, Vendruscolo & Dobson, 2014; Siakallis, Tziakouri‐Shiakalli & Georgiades, 2014), in which normally soluble proteins fold to form unusual, insoluble amyloid fibril forms and may become on‐ and off‐pathway oligomers that are particularly important for cytotoxicity (Ke *et al*., 2017). Their general structural hallmark is a much greater content of β‐sheets than the soluble protein, arranged perpendicular to the fibre axis (Dobson, 2001; Eisenberg & Jucker, 2012; Langkilde *et al*., 2015; Maji *et al*., 2009; Makin *et al*., 2005; Morris & Serpell, 2012; Serpell, 2000; Stromer & Serpell, 2005; Tsemekhman *et al*., 2007; Tycko & Wickner, 2013). Until recently, their insoluble and polymorphic nature made structural studies difficult (Tycko & Wickner, 2013), but recent advances in solid‐state nuclear magnetic resonance (NMR) have led to a general consensus (Colvin *et al*., 2016; Meier & Böckmann, 2015; Tycko, 2016; Wälti *et al*., 2016), at least for the major Aβ peptides. The possibility to form β‐structures in multiple ways underlies the ability of the protein to take different stable conformations (Eichner & Radford, 2011; Eisenberg & Jucker, 2012; Tycko & Wickner, 2013).

Even proteins not normally seen as amyloidogenic or disease‐causing can form amyloids; this is of significance in the storage of biological materials, whose shelf‐life may be shortened as a result [e.g. insulin (Nielsen *et al*., 2001,*b*,*c*; Wang, 2005)]. A similar phase transition to a β‐form is involved in the action of barnacle glue (Nakano & Kamino, 2015), and bacterial inclusion bodies are largely composed of β‐amyloid (de Groot, Sabate & Ventura, 2009). Consequently, understanding this general phenomenon is also important in the field of recombinant protein production.

**Figure 7:**
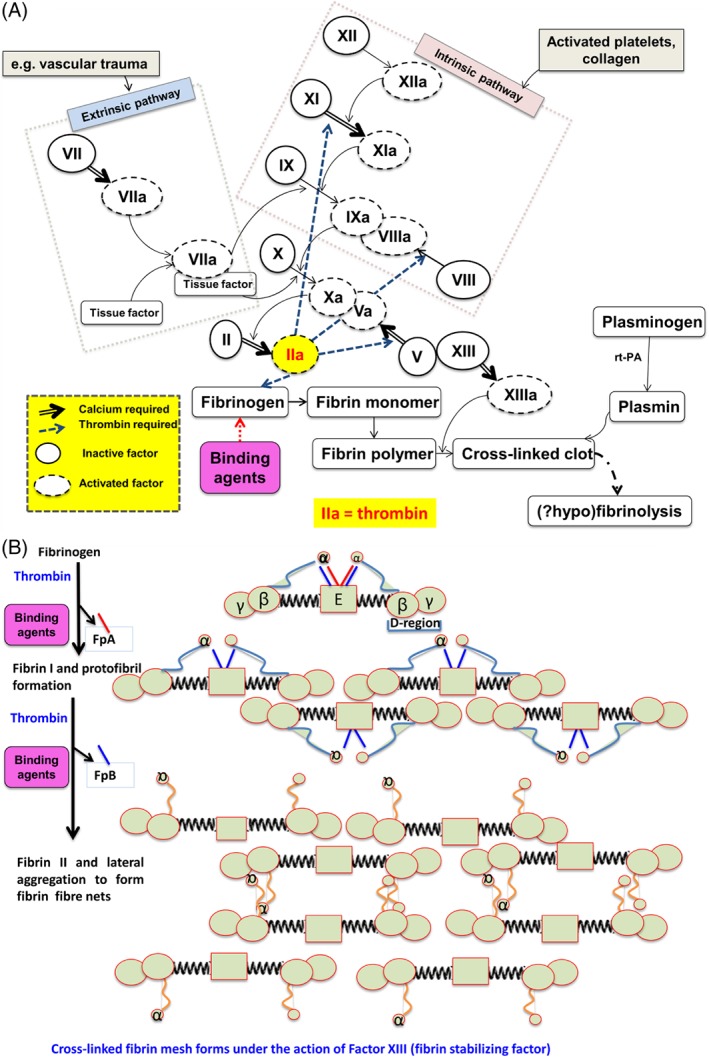
(A) The clotting cascade. Clotting can be activated by either the extrinsic or intrinsic pathway, which converge to a common pathway at factor X, and which ultimately leads to the conversion of prothrombin (factor II) to thrombin that catalyses activation and crosslinking (via factor XIII) of fibrinogen into a fibrin fibre meshwork. Rt‐PA, recombinant tissue plasminogen activator. Redrawn from Kell & Pretorius, 2015b, 2017b). (B) Conversion of soluble fibrinogen molecules to insoluble fibrin fibres during the clotting process (adapted from Kell & Pretorius, 2015b). Fibrinopeptide A and B: FpA and FpB.

**Figure 8:**
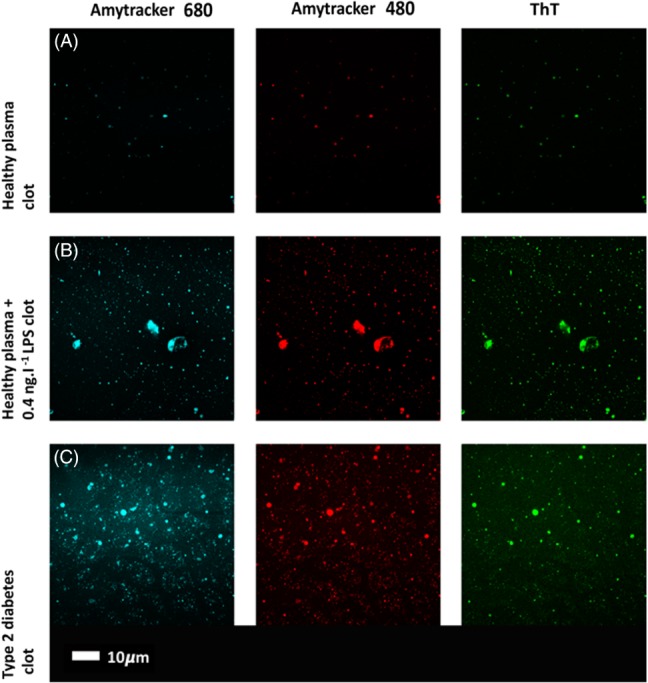
Confocal micrographs of human plasma with added fluorescent markers: Amytracker 480 (blue), Amytracker 680 (red) and Thioflavin T (ThT, green), followed by thrombin to create a fibrin clot. (A) Healthy plasma, (B) healthy plasma after exposure to 0.4 ng l^−1^ lipopolysaccharide (LPS) (Pretorius et al., 2016c); (C) plasma from a patient with type 2 diabetes (Pretorius et al., 2017c).

Blood clotting provides an interesting and novel example (Fig. 7). Scanning electron microscope (SEM) studies showed that blood or plasma clotted in the presence of unliganded iron (Lipinski & Pretorius, 2013b; Pretorius *et al*., 2013a,*b*), formed ‘dense matted deposits’ rather than the normal spaghetti‐ or noodle‐like structures. Similar structures are seen in a variety of disease conditions (e.g. Kell & Pretorius, 2017a; Lipinski & Pretorius, 2013a,*b*; Pretorius, 2011; Pretorius *et al*., 2011, 2016a,*c*, 2015, 2014a, 2017b; Pretorius & Kell, 2014; Pretorius & Oberholzer, 2009). Although a rare mutant in the fibrinogen A chain can cause the molecule to become amyloid (Benson *et al*., 1993; Hamidi Asl *et al*., 1997; Serpell *et al*., 2007), it was not thought that normal fibrin(ogen) would undergo this reaction. However, the observed ‘dense matted deposits’ could be stained with amyloid‐selective fluorogenic stains showing that they were in fact amyloid in nature (Kell & Pretorius, 2017a,*b*; Pretorius *et al*., 2016c, 2017c, 2018*a,b*). This opens up a considerable new biology (Kell & Pretorius, 2015b). A particular feature was that this amyloidogenesis could be induced to occur by the addition of what is stoichiometrically an astonishingly low ratio of bacterial lipopolysaccharide (LPS): fibrinogen, 1:10^8^. Figure 8A and B shows confocal micrographs of healthy (human plasma) before and after exposure to 0.4 ng l^−1^ LPS, followed by the addition of three fluorescent amyloid markers and thrombin. Figure 8C shows a representative clot, with added fluorescent markers, from a type 2 diabetes individual. A similar fluorescent signal to that of healthy plasma with added LPS is present.

As with prions, however, thermodynamics is not an issue (the starting structures are metastable, and the adoption by one protein molecule of an unusual conformation may effectively ‘force’ other molecules of the same type to adjust their conformation. Indeed, one molecule of LPS is sufficient to change the optical properties of millions of molecules of nematic liquid crystal (Lin *et al*., 2011). LPS may also drive the conversion of prions into their amyloid form (Saleem *et al*., 2014). Finally (see Fig. 8), the amyloid structures formed from a given amyloidogenic protein (e.g. fibrinogen) can be highly heterogeneous (Annamalai *et al*., 2016).

### (1) Co‐amyloid formation by fibrin(ogen) and other amyloids

There is considerable evidence that fibrin(ogen) can interact with other amyloid structures (Young *et al*., 2017). The conformation of the fibrin(ogen) involved is unknown, but we suggest that it is almost certainly amyloid as well. Recent studies (e.g. Ahn *et al*., 2017, 2014, 2010; Cortes‐Canteli *et al*., 2010, 2012; Cortes‐Canteli & Strickland, 2009; Zamolodchikov *et al*., 2016; Zamolodchikov & Strickland, 2012) have highlighted its interaction with Aβ peptides in Alzheimer's disease. Here, it is important to recognise that the faster kinetics of a given amyloidogenic process (such as fibrin formation) might accelerate the kinetics of a different amyloid with which it happens to interact, and that this could have important implications for the initiation of overt disease.

Serum amyloid A (SAA) is also an important and potent amyloid. SAA belongs to a family of apolipoproteins associated with high‐density lipoprotein (HDL) in plasma and is an acute‐phase protein synthesised predominantly by the liver (Eklund, Niemi & Kovanen, 2012; Hua *et al*., 2009; Zewinger *et al*., 2015). SAA modulates angiogenesis in many diseases (Lv *et al*., 2016) and is associated with an increase in thrombotic risk (Vitale *et al*., 2014). Traditionally, SAA has been considered to have a key role in the pathogenesis of amyloid A‐type amyloidosis, but it is now known to play a major role in the pathogenesis of chronic inflammatory diseases such as rheumatoid arthritis and atherosclerosis (Eklund *et al*., 2012). SAA has also been found within thrombus material and at sites of ruptured plaques (King, Thompson & Tannock, 2011). Interestingly, SAA expression increases markedly during bacterial infection, tissue damage, and inflammation (Lannergård *et al*., 2008; Li, Ooi & Heng, 2013). During acute inflammation, serum SAA levels may rise up to 1000‐fold, and under these conditions, SAA displaces apolipoprotein A‐I from HDL, thus becoming the major apolipoprotein of circulating HDL3 (Eklund *et al*., 2012). SAA induces the synthesis of several cytokines by binding to and activating cell‐surface receptors, including TLR2 and TLR4, formyl peptide receptor‐like 1 (FPRL1), class B scavenger receptor cluster of differentiation 36 (CD36), and the ATP receptor P2X purinoceptor 7 (P2X7). SAA also activates the inflammasome cascade, which has a key role in immune activation, and has an important role in immunomodulation (Eklund *et al*., 2012). The G‐coupled FPRL‐1 has been demonstrated to mediate SAA‐induced chemotaxis and cytokine release in neutrophils while TLR2 and TLR4 have been identified as novel SAA receptors mediating activities such as pro‐inflammatory cytokine expression in macrophages (Chami *et al*., 2015). SAA also mediates TLR2, and nitric oxide (NO) production *via* mitogen activated protein kinase (MAPK)/ERK signalling pathways in macrophages and TLR4SAA seems to be a ligand for the receptor for advanced glycation end products (RAGE) (Chami *et al*., 2015). Pro‐inflammatory and pro‐thrombotic mediators that are expressed in the presence of SAA include intercellular adhesion molecule 1 (ICAM‐1), vascular cell adhesion molecule 1 (VCAM‐1), IL‐6, IL‐8, monocyte chemotactic protein 1 (MCP‐1) and tissue factor (TF) (Chami *et al*., 2015). SAA can also stimulate vascular cells to express cytokines, chemokines, adhesion molecules and matrix metalloproteinases which are linked to the development of atherosclerosis (King *et al*., 2011).

SAA has also been detected within atherosclerotic lesions and within adipose tissue where it is hypothesised that it may play a contributory role in disease development. In the acute‐phase response, SAA is synthesised by the liver and transported primarily in association with HDL (King *et al*., 2011). However, there might also be localised synthesis of SAA within the vasculature or adipose tissue, where it may play a distinct role in disease development (King *et al*., 2011). Furthermore, SAA can be found in association with apolipoprotein B (apoB)‐containing lipoproteins, in which its biological activity may be different (King *et al*., 2011). Figure 4 includes a brief overview of the activities of SAA when it binds to TLR2 and TLR4.

Although very little information is available regarding the interplay between LPS and SAA, one study suggested that human hepatocytes stimulated by LPS produced SAA (Migita *et al*., 2004). It is well known that SAA has a pro‐thrombotic nature and upregulates a plethora of cytokines (Chami *et al*., 2015). It also interferes with platelet function (Lakota *et al*., 2011) by inhibiting platelet aggregation and modulating platelet adhesion (Sayinalp *et al*., 2004). Furthermore, SAA adheres to human platelets at the arginine‐glycine‐aspartic acid (RGD) adhesion motif‐ and platelet integrin αIIbβ3 receptor (also known as platelet glycoprotein GPllb‐Illa); SAA may therefore play a role in modulating platelet adhesion at vascular injury sites by sharing platelet receptors with other platelet‐adhesive proteins (Urieli‐Shoval *et al*., 2002). SAA consequently plays a fundamental role in creating a pro‐thrombotic environment and hypercoagulation; such an environment is the hallmark of a systemic inflammatory profile.

Many other amyloid proteins can both interact with each other and catalyse further amyloidogenesis (Liu *et al*., 2007; Lundmark *et al*., 2005; Westermark, Lundmark & Westermark, 2009), much as with prions (Kell & Pretorius, 2017a). This phenomenon is essentially what makes them possess what amount to transmissible properties (Lundmark *et al*., 2002; Morales, Callegari & Soto, 2015; Murakami, Ishiguro & Higuchi, 2014; Watts *et al*., 2014; Westermark & Westermark, 2009; Woerman *et al*., 2015). Given that the amyloid form of prion can catalyse its own production, there is now a developing acceptance (e.g. Kell & Pretorius, 2017a; Prusiner, 2012) that prion‐like behaviour and amyloidogenesis are simply two parts of a more general phenomenon. Another consequence is that amyloids can bind molecules such as LPS (Kumar *et al*., 2016).

## XII. STEP 7: DIRECT INDUCTION OF CELL DEATH BY LPS

As well as its role in inducing inflammatory cytokine production, there is some evidence that LPS, albeit commonly bound to proteins that can sequester it, is itself directly cytotoxic [reviewed by Kell & Pretorius (2015a) and Williamson *et al*. (2016)].

## XIII. STEP 8: INFLAMMATION INDUCES COAGULOPATHIES AND THESE CONTRIBUTE TO DISEASE

While we have highlighted amyloid formation as a major part of the dysregulation narrative, inflammation necessarily causes coagulopathies, if only because the concentration of fibrinogen involved (typically 1.5–4 g l^−1^) is associated with a variety of diseases and coagulopathies (Bickel *et al*., 2002; Danesh *et al*., 2005; Davalos & Akassoglou, 2012; Green *et al*., 2010; Zoccali *et al*., 2003).

A general feature of the blood of patients with these chronic inflammatory diseases is that it is both hypercoagulable and hypofibrinolytic (Kell & Pretorius, 2015b); clots form more easily, are stronger, and are less susceptible to proteolysis. The latter is, of course, a particular hallmark of prions (Basu *et al*., 2007; Saá & Cervenakova, 2015; Saleem *et al*., 2014; Silva *et al*., 2015; Woerman *et al*., 2018) and of amyloids generally (Rambaran & Serpell, 2008).

The kinetics of the formation of clots can be studied using thromboelastography to measure clot viscoelastic properties like clot coagulation and fibrinolysis (Pretorius *et al*., 2017d).

## XIV. STEP 9: INDUCTION OF CYTOKINE PRODUCTION BY AMYLOID FORMATION AND VICE VERSA

**Figure 9:**
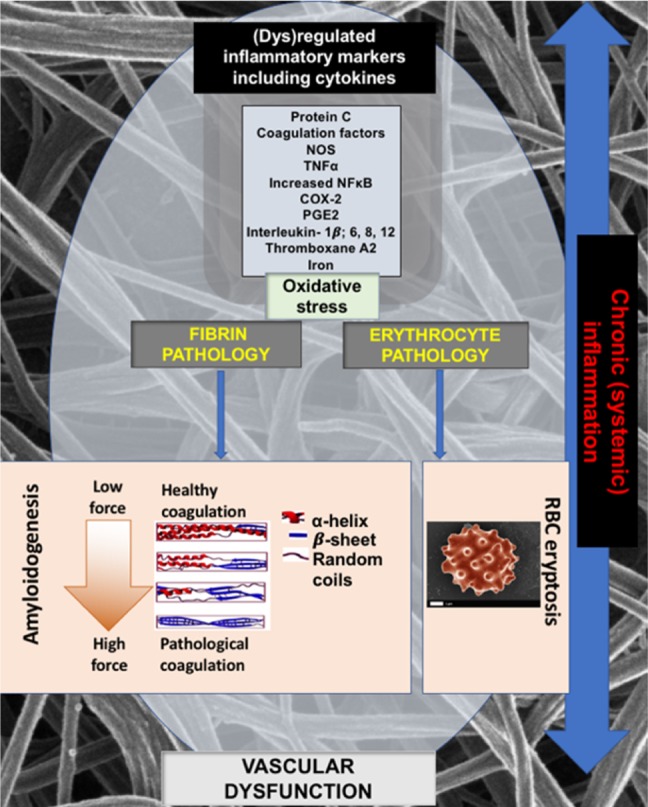
Dysregulation of inflammatory markers, including cytokines and iron, leads to oxidative stress, which in turn causes changes to both fibrin(ogen) and red blood cells (RBCs) visible as amyloidogenesis and eryptosis. Amyloidogenesis and eryptosis both leadsto inflammation but their induction is also enhanced by the presence of inflammation. COX‐2, cyclooxygenase‐2; PGE2, prostaglandin E2; NOS, nitric oxide synthase; TNFα, tumor necrosis factor alpha; thromboxane A2 is a type of thromboxane that is produced by activated platelets.

There is a complex interplay (including positive feedback amplification) between inflammation, cytokine production, amyloid formation and disease (see Fig. 2). A variety of amyloid proteins can themselves induce the formation of inflammatory cytokines (e.g. Gallo *et al*., 2015; Meier *et al*., 2014; Patel *et al*., 2005; Spaulding *et al*., 2015; Westwell‐Roper *et al*., 2011, 2015; Westwell‐Roper, Ehses & Verchere, 2014; Yates *et al*., 2000) and *vice versa* (e.g. Schmidt *et al*., 2017). A simplified example of the inter‐relationship between cytokines, inflammation and visible changes to RBCs and fibrin(ogen) is shown in Fig. 9. Amyloidogenesis and eryptosis are both hallmarks of inflammation and have been associated with vascular dysfunction. However, there is a complex interaction between dysregulated inflammatory markers and the damaging effects of amyloidogenesis and inflammation, and an elementary one‐way approach to the development of inflammation *versus* the upregulation of inflammatory markers will be oversimplifying the complex interactions.

## XV. STEP 10: DIRECT CAUSATION OF DISEASE BY INFLAMMATION?

It is hard to disentangle diseases caused or exacerbated directly by inflammation from those where the mediating agent is explicitly a cytokine. Figure 9 details the complex interactions between dysregulated inflammatory markers as the underlying cause of inflammation but simultaneously subject to inflammation as a catalytic driver of dysregulated inflammatory markers.

## XVI. STEP 11: CELL DEATH (HENCE DISEASE) CAUSED BY AMYLOIDS

Induction of cell death will normally cause disease; for example, if the cells in the substantia nigra pars compacta die the patient will develop Parkinson's, and so on. A great many amyloids have been shown to be cytotoxic, and this is why they are considered in detail herein. What is less clear (Uversky, 2010), although a consensus is now emerging, is which particular class (often equivalent to size) of amyloids are particularly cytotoxic, and what causes this cytotoxicity.

The cytotoxicity of amyloids is well known (e.g. Ahmed *et al*., 2010; Bester *et al*., 2015; Hefti *et al*., 2013; Kayed & Lasagna‐Reeves, 2013; Liu *et al*., 2011; Meyer‐Luehmann *et al*., 2008; Minter, Taylor & Crack, 2016; Miranda *et al*., 2000; Rival *et al*., 2009; Sengupta, Nilson & Kayed, 2016). Interestingly, while larger fibrils are more easily observable microscopically, the modern view is that smaller amyloids [often invisible in conventional SEM, but see Gremer *et al*. (2017)] are more cytotoxic (Aitken *et al*., 2010; Baglioni *et al*., 2006; Bucciantini *et al*., 2002; Dobson, 2013; Fändrich, 2012; Glabe, 2006; Göransson *et al*., 2012; Haass & Selkoe, 2007; Janson *et al*., 1999; Kayed *et al*., 2003; Ke *et al*., 2017; Konarkowska *et al*., 2006; Meier *et al*., 2006; Pillay & Govender, 2013; Stefani, 2012; Trikha & Jeremic, 2013; Xue *et al*., 2009; Xue *et al*., 2010; Zhang *et al*., 2014). However, it would appear that almost all forms of amyloid are cytotoxic [but see Holm *et al*. (2007)] and that they may interconvert. Tests have not yet been performed for the recently discovered (Kell & Pretorius, 2017a; Pretorius *et al*., 2016c, 2018*a,b*) fibrin amyloid, which is considerably larger in fibre diameter than those involved in classical amyloid diseases (Kell & Pretorius, 2017a).

While multiple interactions and processes are likely to be involved, it does seem that membrane interactions are a key event in initiating cytotoxicity (Berthelot, Cullin & Lecomte, 2013; Cao & Raleigh, 2016; Caughey *et al*., 2009; Couthouis *et al*., 2010; Engel *et al*., 2008; Harté *et al*., 2014; Jang *et al*., 2014, 2013; Janson *et al*., 1999; Kegulian *et al*., 2015; Lee *et al*., 2014; Lorenzo *et al*., 1994; Matsuzaki, 2014; Munishkina & Fink, 2007; Okada *et al*., 2016; Suwalsky, Bolognin & Zatta, 2009; Ta *et al*., 2012; Valincius *et al*., 2008), mainly by apoptosis (e.g. Bram *et al*., 2014; Chong, Li & Maiese, 2005; Jang *et al*., 2004; Liu *et al*., 2012; Lorenzo *et al*., 1994; Zhang *et al*., 2010; Zhang *et al*., 2014).

## XVII. HOW GENERAL DO WE CONSIDER THESE MECHANISMS TO BE FOR VARIOUS DISEASES?

The different steps considered herein are entirely generic at a broad level (microbes and their dormant states, iron dysregulation, amyloid formation), with differences only apparent at a finer scale (microbial species and the anatomical location of the various dysregulations). The conditions considered herein are all chronic inflammatory diseases, often with quite slow kinetics, and are all in effect diseases of ageing (e.g. van Beek, Kirkwood & Bassingthwaighte, 2016).

## XVIII. CONCLUSIONS

(1) A systems biology strategy was used to show that chronic, inflammatory diseases have many features in common besides simple inflammation.

(2) The physiological state of most microbes in nature is neither ‘alive’ (immediately culturable on media known to support their growth) nor ‘dead’ (incapable of such replication), but dormant.

(3) The inflammatory features of chronic diseases must have external causes, and we suggest that the chief external causes are (*i*) inoculation by microbes that become and remain dormant, largely because they lack the free iron necessary to replicate, and (*ii*) traumas that induce cell death and the consequent liberation of free iron; these together are sufficient to initiate replication of the microbes.

(4) This replication is accompanied by the production and shedding of potent inflammagens such as lipopolysaccharide or lipoteichoic acid, and this continuing release explains the presence of chronic, low‐grade inflammation.

(5) Recent findings show that tiny amounts of these inflammagens can cause blood to clot into an amyloid form; such amyloid forms are also capable of inducing cell death and thereby exacerbating the release of iron.

(6) Additional to the formal literature that we have reviewed here, it seems to be commonly known that infection is in fact the proximal cause of death in Alzheimer's, Parkinson's, rheumatoid arthritis, multiple sclerosis, etc. It may, for instance, be brought on by the trauma experienced following a fall. Such infections leading to death in chronically ill patients may involve the re‐awakening of dormant bacteria rather than novel exogenous infection. This implies that therapies involving the careful use of anti‐infectives active against dormant microbes could be effective (Coates, Halls & Hu, 2011; Coates & Hu, 2006), as well as the use of nutritional iron chelators (Kell, 2009; Perron & Brumaghim, 2009; Perron *et al*., 2010).

(7) The role of microbes in stomach ulcers is now well established (Marshall, 2002a,*b*, 2003, 2006); here we add to the list of supposedly non‐communicable diseases that can be shown to have a microbial component in their aetiology.
